# Protooncogenic Role of *ARHGAP11A* and *ARHGAP11B* in Invasive Ductal Carcinoma: Two Promising Breast Cancer Biomarkers

**DOI:** 10.1155/2023/8236853

**Published:** 2023-11-23

**Authors:** Y. Naeimzadeh, S. Ilbeigi, H. Dastsooz, M. Rafiee Monjezi, Y. Mansoori, S. M. B. Tabei

**Affiliations:** ^1^School of Advanced Medical Sciences and Technologies, Shiraz University of Medical Sciences, Shiraz, Iran; ^2^Walther-Straub Institute, Ludwig-Maximilians-Universität München, Munich, Germany; ^3^Department of Life Sciences and Systems Biology, University of Turin, Turin, Italy; ^4^Candiolo, C/o IRCCS, IIGM-Italian Institute for Genomic Medicine, Turin, Italy; ^5^Candiolo Cancer (IT), FPO-IRCCS, Candiolo Cancer Institute, Turin, Italy; ^6^Institute for Cardiovascular Prevention, Ludwig-Maximilians-Universität München, Munich, Germany; ^7^Noncommunicable Diseases Research Center, Fasa University of Medical Sciences, Fasa, Iran; ^8^Department of Medical Genetics, School of Medicine, Shiraz University of Medical Sciences, Shiraz, Iran

## Abstract

Invasive duct carcinoma (IDC) is one of the most common types of breast cancer (BC) in women worldwide, with a high risk of malignancy, metastasis, recurrence, and death. So far, molecular patterns among IDC cases have not been fully defined. However, extensive evidence has shown that dysregulated Rho family small GTPases (Rho GTPases) including Rho GTPase activating proteins (RhoGAPs) have important roles in the invasive features of IDCs. In the current study, we analyzed the expression levels of two RhoGAP genes, *ARHGAP11A* and *ARHGAP11B*, in The Cancer Genome Atlas (TCGA) breast cancer (BRCA) and also our 51 IDC tumors compared to their matched normal tissues using quantitative polymerase chain reaction (qPCR). Our TCGA data analysis revealed higher expression of *ARHGAP11A* and *ARHGAP11B* in various cancers comprising BCs. Also, we found correlations between these genes and other genes in TCGA-BRCA. Moreover, our methylation analysis showed that their promotor methylation had a negative correlation with their overexpression. QPCR revealed their significant upregulation in our tumor samples. Furthermore, we found that the expression level of *ARHGAP11A* was considerably lower in women who were breastfeeding. Moreover, it had overexpression in cases who had regular menstrual cycles and early age (younger than 14) at menarche. However, *ARHGAP11B* had a higher expression in HER2-positive tumors versus HER2-positive and ER-positive tumors. Our study found possible protooncogenic roles for these genes and their involvement in IDC pathogenesis and malignancy. Therefore, they can be considered novel prognostic and diagnostic biomarkers for IDC.

## 1. Introduction

Breast cancer (BC) is one of the most common cancer-related deaths among women worldwide [1–4], with approximately 2.3 million new cancer cases and 685,000 deaths reported in 2020 [5]. Generally, the etiology of BC is multifactorial, involving genetic, lifestyle, and environmental factors as well as reproductive behaviors. Several epidemiological studies have shown reproductive factors such as regular menstrual cycles, no history of breastfeeding, early age at menarche, contraceptive use, nulliparity, and first full-term gestation can increase the risk of BCs [5–9]. Based on the expression level of hormone receptor (*HR*) and human epidermal growth factor receptor 2 (*HER2* or *ErbB2*) genes, BCs are divided into different subtypes: basal-like (HR-negative, HER2-negative), normal breast-like (HR-positive/negative, HER2-negative), HER2-enriched (HR-negative, HER2-positive), luminal A (HR-positive, HER2-negative), and luminal B (HR-positive, HER2-positive) [10–14]. Also, there is a valid hormonal differentiation between breast tumors including basal-like and HER2-enriched breast cancers derived from estrogen receptor- (ER-) negative tumors and luminals A and B derived from ER-positive tumors [15]. Moreover, there is a histological classification of breast cancer which includes noninvasive and invasive breast carcinoma. Noninvasive breast carcinoma has 2 subtypes including ductal carcinoma in situ (DCIS) and lobular carcinoma in situ. Invasive breast carcinoma has several subtypes such as invasive (infiltrating) ductal, invasive lobular, ductal/lobular, tubular, mucinous (colloid), and medullary carcinoma [16].

Invasive ductal carcinoma (IDC) and invasive lobular carcinoma (ILC) are the most common variants of basal-like breast carcinoma (BLBC) [12, 17]. IDC usually starts from the milk ducts, develops to other parts of the breast such as fatty tissue, also known as infiltrating carcinoma, and accounts for about 80% of all invasive BC, including TNBCs with a solid growth pattern and a high risk of malignancy, recurrence, and death [17–20]. BLBCs are related to BRCA1 germ-line mutation tumors and progress among BRCA1 mutation carriers [15, 17, 21]. The relevant molecular mechanism of the IDC is not elucidated, and thus, the optimal management of the IDC has become increasingly complex [20]. Further investigations are needed to identify effective approaches for the diagnosis and treatment of IDC.

Already, several various molecules have been identified which play important roles in the initiation and development of BCs. One of the important members of the Ras superfamily is the Rho family of small GTPases (Rho GTPases) which are intracellular signaling molecules and are responsible for regulating cellular functions like cell cycle progression, cytokinesis, polarity, cell migration, adhesion, and invasion. So, they have key roles in cancer metastasis [22–24]. About 22 members of them were identified which are classified into 3 groups: *RhoA*, *RAC1*, and *CDC42* [25, 26]. Their functions are controlled by switching Rho GTPases between active (GTP-bound) and inactive (GDP-bound) forms [24, 27]. In a normal situation, the function of these proteins is regulated by 3 classes of molecules including GTPase-activating proteins (GAPs), guanine nucleotide exchange factors (GEFs), and guanine nucleotide dissociation inhibitors (GDIs) [10, 23, 24]. GAPs boost the exchange of GTP to GDP to turn GTPase into the inactive form, GEFs promote the catalysis of GDP to GTP that activates GTPase, and GDIs separate GTPases in the GDP-bound form to keep the GTPase in an inactive form [28]. *ARHGAP11A* (Rho GTPase activating protein 11A) encodes a 1023-aa protein with RhoGAP activity located on chromosome 15 (i.e., 15q13.3) [23, 29–31]. Recent studies demonstrate that *ARHGAP11A* has a higher expression in BLBCs than the other subtypes [32]. Also, a chromosomal deletion which includes *ARHGAP11A* is one of the reasons for the Prader-Willi syndrome [33], as well as *ARHGAP11B* is located on chromosome 15 (15q13.2). This gene was derived from the partial duplication of *ARHGAP11A* after the human lineage was possibly separated from the chimpanzee lineage [34–36]. It encodes a truncated protein that is capable of promoting the development of basal progenitors (BPs) and expanding the brain neocortex, but, unlike the previous gene, RhoGAP activity was not observed in this gene [29, 30]. Since the RhoGAPs have a key role in dynamic changes in cell shape and metastasis in invasive breast tumors including TNBCs [37], we decided to investigate the expression of two paralog genes (*ARHGAP11A* and *ARHGAP11B*) in IDC cases. Also, their possible correlation with multiple reproductive factors may be used as biomarkers for prognosis, diagnosis, or therapeutic targets for IDC patients.

## 2. Materials and Methods

### 2.1. Bioinformatic Analysis

To find the role of *ARHGAP11A* and *ARHGAP11B* in breast cancer, we first analyzed their mRNA expression in TCGA-BRCA using the TACCO webserver (http://tacco.life.nctu.edu.tw). Then, using the LinkedOmics webserver (http://www.linkedomics.org/login.php), we investigated their promoter methylation to identify a possible correlation between their altered expression and the methylation in TCGA-BRCA. In this methylation analysis, we considered methylation Meth450 Gene Firehose Methylation Preprocessor data (units: beta value). Regarding their mRNA expression to correlate the promoter methylation, we selected RNAseq HiSeq Gene Firehose RSEM log2. For this analysis, the Spearman correlation was considered on the LinkedOmics webserver. Moreover, in another way, we used other webservers to look for this correlation in relation to all CpG island positions related to their transcripts with higher expression specific to breast cancer. To do this, firstly, using GEPIA2 (http://gepia2.cancer-pku.cn), we looked for ARHGAP11A and ARHGAP11B isoform expression in TCGA-BRCA and considered these specific isoforms for further analysis in another way of methylation/gene expression analysis using Shiny Methylation Analysis Resource Tool (SMART) (http://www.bioinfo-zs.com/smartapp/). Moreover, we looked for positively and negatively coexpressed genes with *ARHGAP11A* and *ARHGAP11B* using RNAseq HiSeq Gene Firehose RSEM log2 data from the LinkedOmics webserver (http://www.linkedomics.org/login.php). Next, using the data of coexpressed genes, we investigated gene set enrichment analysis (GSEA), including biological processes (BP) and KEGG pathways related to these genes by this webserver. Finally, we analyzed their expression across cancer stages and other patients' statuses in TCGA-BRCA using the UALCAN webserver (http://ualcan.path.uab.edu/index.html). Moreover, using the Kaplan Meier plotter (https://kmplot.com/analysis/index.php?p=background), an online webserver, we showed the relationship between ARHGAP11A and ARHGAP11B and the overall survival (OS) possibility in different types of breast cancer.

### 2.2. Study Population

A total of 51 invasive ductal carcinoma breast cancer patients who had received no chemotherapy or radiotherapy were included in this study. All subjects were selected from individuals referred to Faghihi Hospital, affiliated with Shiraz University of Medical Sciences. Written informed consent was obtained from all patients, and the study was approved by the Ethics Committee of Shiraz University of Medical Sciences, Shiraz, Iran. These samples and their clinical data were also used for our previous study [20]. The demographic, reproductive, and clinical information as well as the pathological data of tumor samples were obtained from each participant in the current study. Pathology data, including histological type and grade (ER, PR, and HER2 status), were evaluated by two academic pathologists based on American Society for Clinical Oncology (ASCO) guidelines.  Table 1 shows the characteristics of the study participants. In terms of age, the participants were 27-68 years old (mean ± SD: .... ± ....). Fifteen (~30%) participants were aged < 40 years and 36 (~70%) were ≥40 years old. Among the 51 participants, 36 (~70%) were parous (P) and 15 (~30%) were nulliparous. Regarding the menopausal status, 4 (~8%) participants were premenopausal and 47 (~92%) were in postmenopausal status. Moreover, the participants were divided into 2 subgroups based on the age at menarche of <14 or ≥14 years. The age at the first full-term pregnancy and breastfeeding duration were also recorded. Parous participants were divided into 2 subgroups according to the age at the first full-term pregnancy of <25 or ≥25 years.

### 2.3. Breast Tissue Sampling

The tumor samples were precisely taken from the central part of the solid tumor to avoid adjacent normal tissue contamination, and again, for normal tissue, sampling was done from the farthest part of the solid tumor. The tissues had been snap-frozen in liquid nitrogen immediately and stored at -80°C until RNA extraction.

### 2.4. Estrogen Receptor (ER), Progesterone Receptor (PR), and HER2 Determination

The ER, PR, and HER2 status were determined according to the patient's histopathological data, following immunohistochemistry (IHC) staining. If ≥1% of tumor cells show positive ER/PR staining, the ER/PR interpretation is positive. As well, the IHC HER2 score of 3+ was considered positive.

### 2.5. RNA Isolation and Real Time-PCR

Total RNA was extracted from all tissue samples and cell lines using TRIzol as recommended by the manufacturer (Life Technologies, Carlsbad, CA), and the final concentration was quantified using a NanoDrop at 260 and 280 nm. The range of purity (both 260/280 and 260/230 nm) of RNA was 2, and we used the RNA concentration of 500 ng. The RNA integrity was confirmed by gel electrophoresis, and to remove the probable DNA contamination, the total RNA was treated with DNase (Takara Bio Inc., Otsu, Japan) according to the manufacturer's instructions. The cDNA synthesis was carried out using approximately 500 ng of total RNA with the Prime Script-RT kit (Takara, Japan) according to the manufacturer's protocol. Next, real-time PCR was carried out in a QuantStudio™ 3 system (Applied Biosystems, USA, Thermo Fisher Scientific) according to the manufacturer's protocol for the SYBR Premix Ex Taq II kit (Takara, Japan). Real-time specific primer pairs used were as follows: *ARHGAP11A*-forward: GAGACGGTTTCACCGTGTTAG, *ARHGAP11A*-reverse: GGTGGCTCATGCCTGTAATC; *ARHGAP11B*-forward: GCATCCTGCCTCAGAGTTATC, *ARHGAP11B*-reverse: CGGACACCCTTCACCTTAATAC; and *B2M*-forward: AGATGAGTATGCCTGCCGTG, *B2M*-reverse: GCGGCATCTTCAAACCTCCA.

For each reaction, 10 *μ*l SYBR® Green Master Mix with 0.8 *μ*l (40 nM) primer 1 and 0.8 *μ*l (40 nM) primer 2 with 2 *μ*l was used, and the final volume was adjusted to a total of 18 *μ*l using dH2O. All the reactions were carried out in triplicate. The real-time PCR was performed under the following conditions: 30 s at 95°C, followed by 40 repetitive cycles of 30 s at 95°C, and then 30 s at 60°C. No template controls (NTCs) were included in each run. The relative mRNA expression levels of *ARHGAP11B* and *ARHGAP11A* were normalized to the *β*2 microglobulin expression level as a housekeeping gene. The expression level (i.e., fold change) for each gene was calculated using the 2^−ΔΔCT^ method.

### 2.6. Statistical Analysis

The data are presented as the standard deviation and mean. qPCR data were analyzed by the unpaired *t*-test and the Mann-Whitney test. The comparison of gene expression among the subgroups was performed by a *t*-test or ANOVA. Subsequently, we compared the expression levels of *ARHGAP11A* and *ARHGAP11B* between the subgroups via nonparametric tests using Kruskal-Wallis and Mann-Whitney. To investigate the correlation between the expression of the two genes and their promoter methylation in TCGA BRCA, the Spearman correlation was considered using the LinkedOmics webserver. All statistical analyses were done by SPSS version 20.0 software (IBM, Carlsbad, CA, USA). We considered *P* values < 0.05 to be statistically significant.

## 3. Results

### 3.1. Expression of *ARHGAP11A* and *ARHGAP11B* in TCGA Cancers

Our TCGA data analysis using the TACCO webserver (http://tacco.life.nctu.edu.tw) revealed that *ARHGAP11A* and *ARHGAP11B* were among the upregulated genes in BRCA. Moreover, our data found that *ARHGAP11A* and *ARHGAP11B* were also overexpressed in almost all TCGA cancers (Tables 2 and 3), representing their possible protooncogenic role.

**Figure 1:**
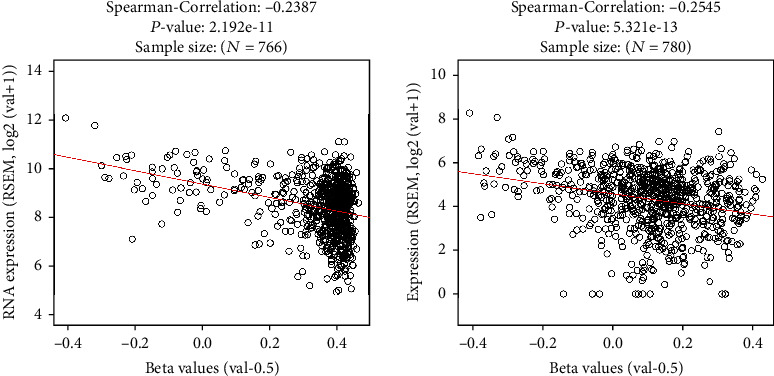
Promoter methylation analysis of *ARHGAP11A* and *ARHGAP11B*. Data shows the promoter hypomethylation of these genes.

Then, we investigated the correlation between their overexpression and methylation using the LinkedOmics webserver. Our methylation analysis revealed that the promoter methylation of *ARHGAP11A* and *ARHGAP11B* had a significant negative correlation with their upregulation (*ARHGAP11A*: Spearman's correlation: -0.24, *P* value: 2.192*e*‐11; *ARHGAP11B*: Spearman's correlation: -0.25, *P* value: 5.321*e*‐13, Figure 1). Moreover, to strengthen this correlation, using another webserver, we analyzed the correlation between the RNA expression of these genes and methylation status. We found that the ENST00000361627.7 isoform of ARHGAP11A and the ENST00000602616.6 isoform of ARHGAP11B showed higher expression in TCGA-BRCA. Therefore, we considered these isoforms for the correlation between RNA expression and methylation of CpG island positions in these genes. As seen in supplementary file S1, using SMART, these transcripts showed negative correlation expression to the methylation status of the genes in the aggregation method (the mean/median methylation for all the individual CpGs selected).

**Figure 2:**
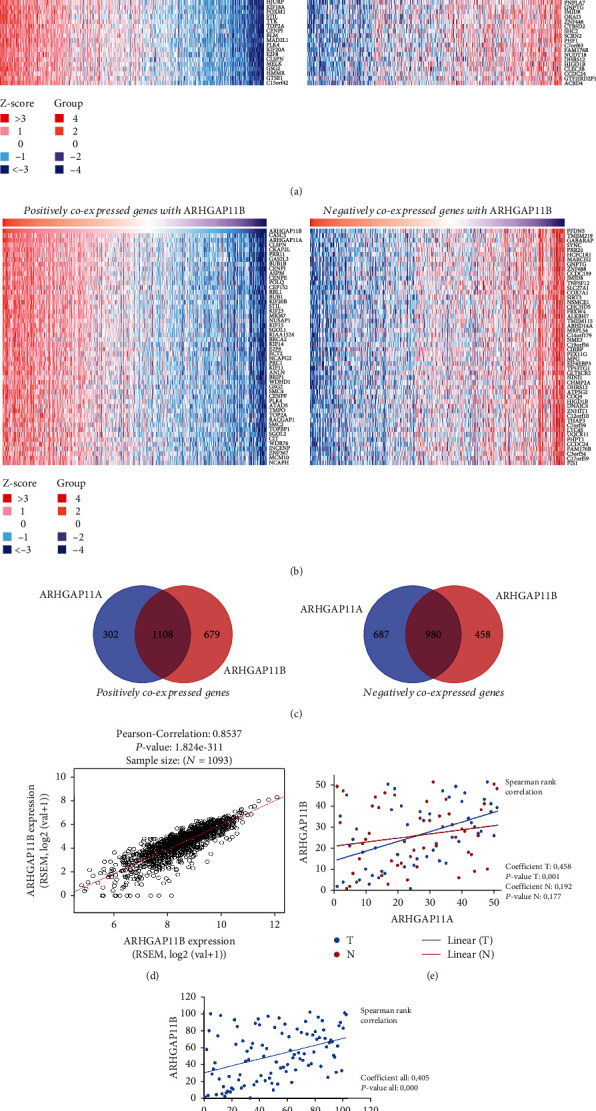
(a, b) Positively and negatively coexpressed genes with ARHGAP11A and ARHGAP11B in TCGA-BRCA, respectively. (c) Venn diagram represents the number of coexpressed genes common between and specific to these genes. (d) ARHGAP11A and B mRNA expression correlation in TCGA-BRCA. (e) Positive correlation between these genes in our 51 tumor samples but not in their normal adjacent to these tissues. (f) Positive correlation between these genes in all samples together.

To find coexpressed genes for *ARHGAP11A* and *ARHGAP11B* in TCGA-BRCA, we used LinkedOmics and identified several shared genes with positive and negative mRNA expression correlations to these two genes (Figures 2(a) and 2(b)).  Figure 2(c) represents the number of coexpressed genes which were common in or specific to *ARHGAP11A* and *ARHGAP11B*. We only provided coexpressed genes with an R correlation score > 0.3 (based on the previously proposed middle positive R correlation score [38]) and an adj *P* value < 0.05 and also prepared the Venn diagram using online Venn diagram tools (https://bioinformatics.psb.ugent.be/webtools/Venn/). The Venn diagram represents that the number of common coexpressed genes is greater than specific ones for both negatively and positively coexpressed genes, with more genes for positively coexpressed genes (Figure 2(c)). It should be noted that both genes showed positive correlation expression with each other (Figure 2(d)). Moreover, performing correlation analysis for these two genes in our 51 tumor samples showed their positive correlation in the tumor samples (Spearman's coefficient: 0.458 and *P* value: 0.001) but not in their matched normal tissues (Spearman's coefficient: 0.192 and *P* value: 0.177) (Figure 2(e)). However, when we performed correlation analysis for all samples altogether, they were correlated (Spearman's coefficient: 0.405 and *P* value: 0.000) (Figure 2(f)).

Among the top 100 coexpressed genes with *ARHGAP11A* and *ARHGAP11B,* there were several genes in common, and most of them are involved in breast cancer, including (genes that contributed to breast cancer are shown with their corresponding references) *BUB1B* [38, 39], *BUB1* [38, 40], *KIF20A* [38, 41], ASPM [42], *TOP2A* [43], *CKAP2L* [44], *KIF23* [45], *NUSAP1* [46], *PRC1*, *SGOL1* [47], *KIF11* [48], *MKI67* [49], *NCAPH* [50], *POLQ* [51], *RACGAP1* [52], *KIF14* [53], *KIF15* [54], *MCM10* [55], *CENPF* [56], *CASC5*, *STIL*, *CENPI*, *PLK4* [57], *E2F8* [58], *CLSPN* [59], and *GSG2* [60].

The presence of commonly coexpressed genes for both *ARHGAP11A* and *ARHGAP11B* indicates common pathways for these two genes. Therefore, we looked for GSEA using LinkedOmics for GO: BP terms and KEGG pathways, and the results revealed some similar cancer processes and pathways such as cell cycle, cellular senescence, DNA replication, recombination, cancer, and among others (Supplementary files S2 and S3). Moreover, we identified different BP terms and KEGG pathways specific to *ARHGAP11A* and *ARHGAP11B*, as well as common ones given in Supplementary file S4.

**Figure 3:**
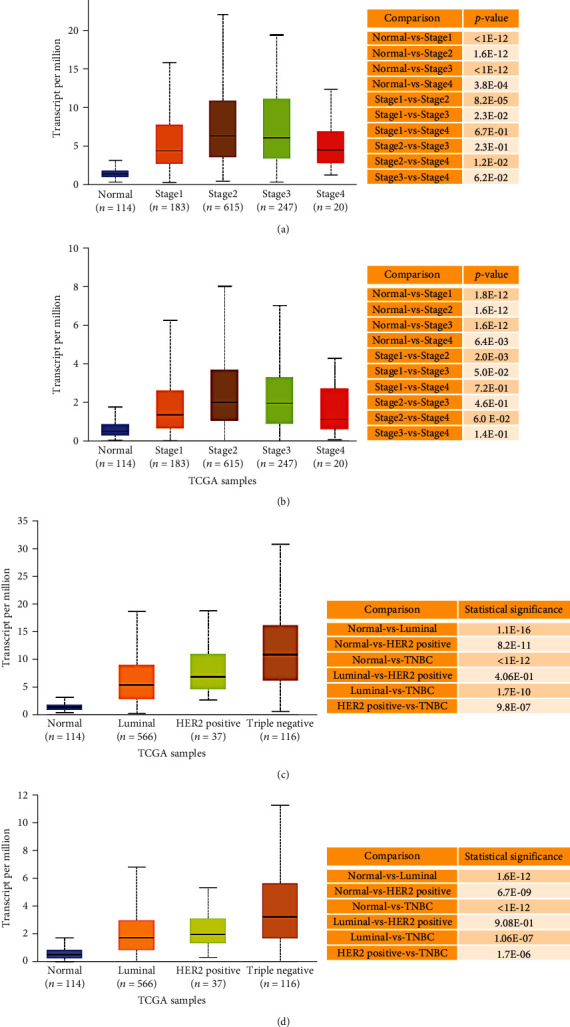
(a, b) *ARHGAP11A* and *ARHGAP11B* mRNA expression in BRCA based on cancer stages, respectively. (c, d) *ARHGAP11A* and *ARHGAP11B* mRNA expression in BRCA subclasses, respectively.

**Figure 4:**
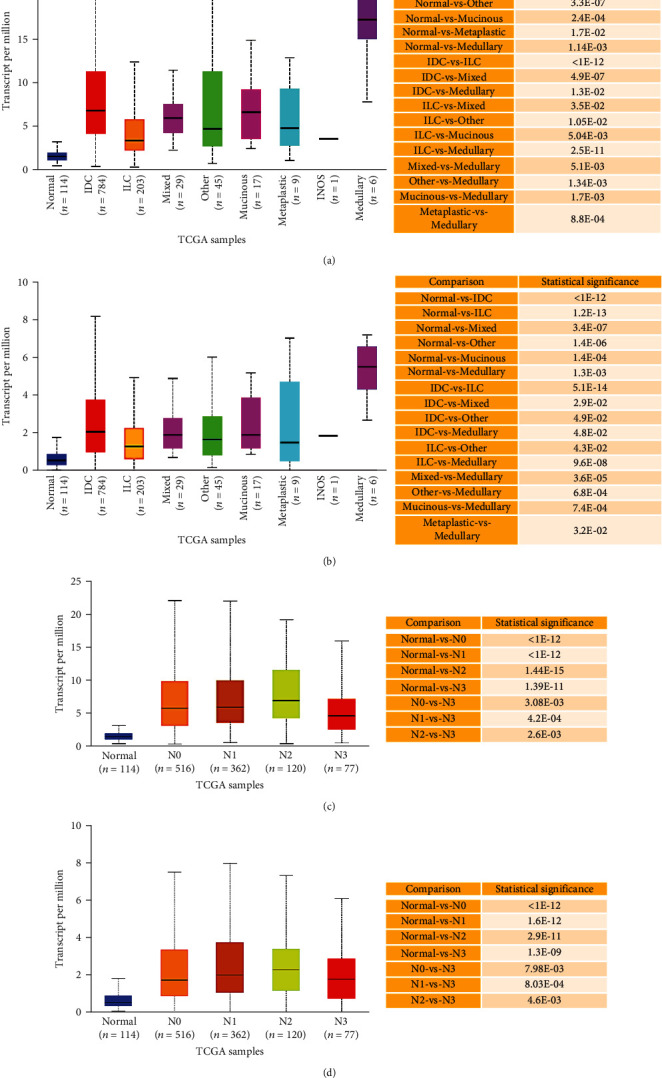
(a, b) *ARHGAP11A* and *ARHGAP11B* mRNA expression in BRCA based on histological subtypes, respectively. (c, d) *ARHGAP11A* and *ARHGAP11B* mRNA expression in BRCA based on nodal metastasis status, respectively. IDC: infiltrating ductal carcinoma; mixed: mixed histology; medullary: medullary carcinoma; INOS: infiltrating carcinoma NOS; ILC: infiltrating lobular carcinoma; mucinous: mucinous carcinoma; metaplastic: metaplastic carcinoma.

Regarding TCGA-BRCA tumor stages, our analysis revealed that *ARHGAP11A* and *ARHGAP11B* showed overexpression across BRCA cancer stages, indicating their role in tumorigenesis and invasion (Figures 3(a) and 3(b)). Moreover, regarding BRCA subclasses, our data found that their expressions were significantly higher in triple-negative, luminal, and HER2-positive subclasses compared to their matched normal tissues. Furthermore, our results identified their significantly higher expression in TNBC compared to luminal and HER2-positive (Figures 3(c) and 3(d)). In addition, our TCGA data analysis showed that all BRCA histological subtypes had higher expression of these two genes compared to their normal tissues, with a significant number of cases (i.e., 784) and the most significant higher expression of these genes for IDC (Figures 4(a) and 4(b)).

In relation to the nodal metastasis statuses, we found that these genes had higher expression in all N0, N1, N2, and N3 tissues compared to normal tissues. It seems that their higher expression is important to progress from normal to nodal metastasis (Figures 4(c) and 4(d)).

Regarding the expression of these genes among different races, we analyzed their expression from TCGA-BRCA in different races including Caucasian, African-American, and Asian. The data showed a higher expression of these genes in all races compared to normal, but there was no significant difference when each race was compared to another one (supplementary file S5).

In our study, using the Kaplan-Meier plotter, we also investigated the correlation between the expression of ARHGAP11A and ARHGAP11B and OS possibility in different types of breast cancer. As shown in the supplementary files S6 and 7, respectively, when we considered all lymph node, ER, PGR, HER2, and KI67 statuses, all Nottingham histologic grades, and all PAM50 subtypes altogether, in our analysis, results showed that higher expression of the ARHGAP11A gene had a shorter OS time and worse OS prognosis, but higher expression of the ARHGAP11B gene revealed a longer OS time and better OS prognosis.

However, in relation to ARHGAP11B, with the selection of one basal subtype and HER2, its higher expression had a shorter OS time and worse OS prognosis, but with the selection of only luminal B, its higher expression showed a longer OS time and better OS prognosis.

However, regarding ARHGAP11A, when we selected one of the PAM50 subtypes (normal, basal, luminal A, and luminal B), individually, only in the luminal B subtype did the higher expression of ARHGAP11A reveal a better OS time and a better OS prognosis, but other subtypes did not show a significant correlation between its expression and OS.

Therefore, this data showed that the OS could be correlated with different breast cancer subtypes for either a better or worse prognosis, and it seems that higher expression of both genes in the luminal B subtype is correlated with a better prognosis, but other subtypes revealed a worse prognosis or no significant correlations.

### 3.2. Expression Levels of *ARHGAP11A* and *ARHGAP11B* in BC Tissues

**Figure 5:**
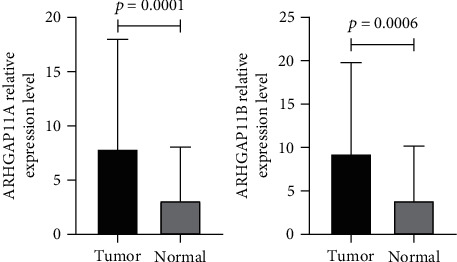
The expression levels of *ARHGAP11A* and *ARHGAP11B* genes in IDC tumors and matched normal tissues.

We also investigated the expression level of these genes in our 51 breast tumor tissues and their adjacent normal tissues using qPCR. Our results demonstrated that the expression level of *ARHGAP11A* (*P* = 0.0001) had remarkable overexpression in tumor tissues compared to normal tissues. Furthermore, it was revealed that *ARHGAP11B* (*P* = 0.0006) was significantly upregulated in tumor samples versus normal tissues. The results of the relative expression levels of *ARHGAP11A* and *ARHGAP11B* in tumor samples compared with normal tissues are shown in Figure 5.

### 3.3. The Expression Level of *ARHGAP11A* and *ARHGAP11B* regarding the Reproductive and Clinicopathological Characteristics

The comparison of the expression levels of the two genes in various subgroups of the patients regarding several reproductive and clinicopathological properties of the subjects was performed. The results of the *t*-test demonstrated that the expression level of *ARHGAP11A* was considerably higher in the subgroup of participants who had regular menstrual cycles (*P*: 0.013). Also, the expression level of *ARHGAP11A* was significantly lower in patients who had long-term breastfeeding (*P*: 0.034). Furthermore, this gene had overexpression in patients with an age at menarche of <14 years compared to participants with an age at menarche of ≥14 years (*P*: 0.041). No considerable differences were observed for the other variables in the expression level of this gene among the subgroups (Table 1).

Also, the results of the *t*-test in HR and HER2 showed that the level of expression of *ARHGAPP11B* is higher in HER2-positive (*P*: 0.031) and ER-positive subgroups (*P*: 0.041). Any significant differences were not identified for other variables among the subgroups (Table 4).

## 4. Discussion

IDC as a major part of BLBC with highly aggressive features overlaps by up to 80% with TNBCs [17, 23]. Most of our results are consistent with previous studies in this field. However, this is the first time that *ARHGAP11A* and *ARHGAP11B* have been investigated in IDC patients in terms of reproductive and clinicopathological factors. As mentioned above, in our study, we first performed a bioinformatic investigation regarding the role of these two RhoGAP paralog genes (i.e., *ARHGAP11A* and *ARHGAP11B*) in BC and different cancers and their correlations with each other and with other genes in different cancers. Then, the promoter methylation of both genes was investigated. Next, we compared the expression level of two RhoGAP paralog genes (i.e., *ARHGAP11A* and *ARHGAP11B*) in tumor and normal breast samples of IDC cases and evaluated them in several reproductive, demographic, and clinicopathological factors among women with IDC.

Our reproductive investigations demonstrated that *ARHGAP11A* had lower expression in women who have had a long period(s) of breastfeeding than in nonlactating women. Nowadays, the influence of the long period of breastfeeding against invasive BCs is supported by several documented studies. For instance, a previous study among postmenopausal women revealed that the risk of TNBC is 50% lower in parous women who were lactated at least for 6 months than in parous women without a history of breastfeeding [61]. A similar consistent finding was manifested that breastfeeding has an inverse relationship with TNBCs and luminal BCs including luminal A [11, 62] and a 50% decline in TNBC odds in young females who had been breastfed for more than 12 months [63]. According to another research, breastfeeding can diminish the risk of some types of BC, such as BRCA1-associated breast cancer (e.g., BLBCs), among women who have had two or more years of lactation [64]. Also, it was demonstrated that the risk of BC among parous females with exclusive breastfeeding histories is significantly lower compared with parous females without experience of exclusive breastfeeding [65]. A possible explanation is that the whole number of menstrual cycles is reduced by breastfeeding [66]. Indeed, women who did not experience breastfeeding are at higher risk of breast cancer because they are exposed to a rise and fall in estrogen and progesterone levels in their lifetime more than other females, thanks to more menstrual cycle experiences [66]. Interestingly, in addition to BCs, breastfeeding can reduce the risk of endometrial cancer, one of the most common cancers, among women with similar hormonal reasons, especially estrogen [67]. It was manifested that in the presence of estrogen exposure, HR-negative cells are stimulated to proliferate by paracrine signals which are produced by neighboring HR-positive progenitor cells and lead to the development of some BCs such as TNBCs [11]. Also, there is an association between breastfeeding and constant alteration in the molecular breast cell histology determined by terminal duct lobular unit involution, which might decrease the risk of BC, particularly BLBC. It seems that this molecular involution which is regulated by STAT3 signaling, has a positive effect on the mammary gland during the lactation period [11, 62].

Moreover, this research showed that *ARHGAP11A* had overexpression in participants with an age at menarche of <14 years compared to participants with a late age at menarche. Nowadays, early age at menarche is well known as a risk factor for BC development, and several documented studies have revealed that this factor has a great effect on the development of BLBCs [68, 69]. Although there are some contradictory observations [62, 68, 70], they mentioned early menarche as a risk factor for TNBCs [62, 68], and other studies have shown that there is a connection between early menarche and late menopause with ovarian cancers and BCs [66, 71]. A possible hypothesis is that the early proliferation of mammary gland cells is stimulated by early menarche; because of early exposure to high hormonal levels by experiencing more menstrual cycle oscillations in their lifetime, these females are more vulnerable to experiencing BC than other females [72]. In fact, early age at menarche is related to an early enhancement of follicle-stimulating hormone in serum, higher circulating estradiol condensation before and for some years after first menstruation, and the early beginning of ovulatory cycles. These occurrences proposed that this hormonal situation in the early ages of maturation might lead to BC [68].

Also, our results revealed that *ARHGAP11A* has overexpression in women who had regular menstrual cycles rather than participants with irregular menstrual cycles. Previous studies have shown that women who had short, regular, and more menstrual cycles before the first full-term gestation are more susceptible to developing BC compared to women without a history of BC or with benign breast disease. It was shown that at the end of the reproductive life of women who had irregular menstrual cycles, benign breast disease is more prevalent. Women with cystic ovarian and breast cancer due to irregular menstrual cycles have a lower risk of BC [73]. Indeed, in the long run, frequent falls and rises of estrogen and progesterone levels pending a large number of menstrual cycles would provide the situations for changing the fate of the cells by stimulating random mutations and genome instability which may eventually lead to BC. In fact, the hormonal changes mentioned above that occur each month with each menstrual cycle can affect the MaSCs (i.e., mammary stem cells) that might enhance the risk of tumor initiation by random genetic errors. A lack of coordination between the proliferation of mammary epithelial cells and apoptosis would provide an ideal situation for breast tumor cell progression [72].

According to all previous supportive evidence, women who have early age at the first menstruation (before age 12), women with regular menstrual cycles, women who go through menopause later than others (after age 55), and women who did not experience breastfeeding are at higher risk of breast cancer due to similar hormonal etiology [66]. In this research, we figured out that *ARHGAP11A* has an overexpression in relation to the above reproductive factors. Moreover, our bioinformatic studies have displayed that this gene and its paralog gene *ARHGAP11B* are involved in the main biological processes and pathways and have key roles in tumorigenesis, invasion, and nodal metastasis in BCs. So far, many genes have been identified as responsible for the initiation and development of BCs which have tumor-suppressive or oncogenic roles, and recent advanced studies have made considerable developments in the detection of molecular mechanisms related to the aggressiveness and pathogenesis of BCs. But it seems that the molecular etiology of BC is very sophisticated and heterogeneous, with many unknown aspects. Therefore, our results related to these two genes can provide a relevant contribution to this field of study.

**Figure 6:**
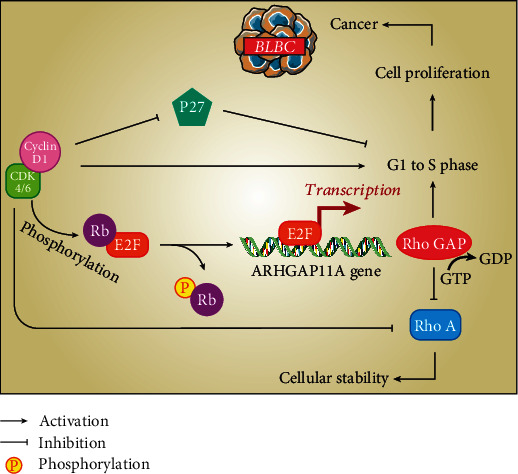
The molecular mechanism of the oncogenic role of the *ARHGAP11A* gene in BLBC.

Generally, mutations in Rho GTPases are scarcely responsible for cancer. Instead, dysregulation of the expression and/or activity of Rho GTPases in cancer is more common [10, 23]. However, GEFs usually have an oncogenic role, and GAPs and GDIs have tumor suppressor activity in various cancers [28]. Determining the role of RhoGAPs in the cancer process is almost complicated thanks to their different activities and expression levels in various cancers. A previous investigation has demonstrated some RhoGAPs (e.g., DLC1) have a tumor-suppressive role in cancers due to deletion or loss of expression [23]. In these cases, in reaction to DNA damage, the encoded protein of ARHGAP binds to P53 (a tumor suppressor) by the RhoGAP domain and stimulates apoptosis. This issue is a justification for the deletion of RhoGAPs in several cancers [74, 75]. According to a bioinformatic study, *ARHGAP6*, *10*, *14*, *19*, *23*, and *24* had the lowest expression, and *ARHGAP11*, *15*, *18*, and *30* had overexpression in BC patients compared to normal persons [1]. Several studies revealed that, in addition to various types of BCs, especially BLBCs, *ARHGAP11A* has a high expression level in human glioblastoma, colon, lung, hepatocellular, and gastric cancers [31, 74, 76]. Also, it was revealed that applying interfering RNA- (iRNA-) based suppression to *ARHGAP11A* can decrease the aggressive properties of several cancers which can reveal the oncogenic role of this RhoGAP [74]. The molecular mechanism of this phenomenon is displayed below (Figure 6). While genomic instability in BLBCs due to the inactivation of Rb (a tumor suppressor) had been reported before [12], transcription and expression of *ARHGAP11A* are related to *E2F1* and have overexpression in the S phase of the cell cycle by Rb blocking. Indeed, *ARHGAP11A* is one of the upregulated functional gene networks as a target for *E2F1*. Thus, phosphorylation and inhibition of Rb that occur with cyclin D1-CDK4/6 [77–80] might result in the abnormal activity of E2Fs and can lead to overexpression of *ARHGAP11A* which ultimately stimulates cell proliferation via cellular movement in various cancers, especially BC [32, 74, 80]. Moreover, Lawson and Der have indicated in BLBC that cell cycle arrest in the G1 phase occurred by upregulation of p27 (a CDK inhibitor) which is the result of the *ARHGAP11A* downregulation [23], and the overexpression of *ARHGAP11A* in the G1 to S/G2/M phase transition might occur through the p27 reduction and cyclin D1 enhancement. These phenomena can lead to RhoA inhibition and reduce the formation of stress fibers and focal adhesions which leads to stimulating cell migration and invasive features in breast cells, especially BLBCs [10, 23, 31, 32, 74, 81]. Generally, RhoA has a complicated role in various cancer cells; even though some evidence has revealed that RhoA has an oncogenic role in some BC cell lines such as MC7 [28, 82], lots of studies have shown that RhoA inhibition can stimulate cell migration and lead to metastasis in BLBCs and TNBC cell lines like MDA-MB-231, which overall indicate the tumor suppressive role of RhoA in most BC cells [23, 28, 83]. Also, it was demonstrated that not only overexpression of *ARHGAP11A* leads to cell proliferation in BLBC but also alteration of the normal cells into the cancer phenotype [23].

Moreover, our TCGA analysis showed that *ARHGAP11A* has an oncogenic role in various cancers such as lung, colon, kidney, and BC, especially in TNBCs, and our TCGA data analysis revealed that all BRCA histological subtypes had overexpression in 787 IDC cases. It seems that this gene may be specific to this BC subtype. According to our bioinformatic and reproductive studies among IDC patients and approval evidence in BLBC tumors, it can be concluded that *ARHGAP11A* has an oncogenic role in IDC as a major subgroup of BLBCs.

For the second gene, *ARHGAP11B*, our results showed that this gene had overexpression in ER-positive and HER2-positive tumor samples. The overexpression of this gene in BLBC was shown in one of the previous researches [10]. Also, our bioinformatic analysis revealed that, in addition to TNBCs, this gene and its paralog (*ARHGAP11A*) had considerable overexpression in HER2-positive subclasses. Also, this gene like its own paralog gene is upregulated in various cancers such as kidney, lung, stomach, and BC. As mentioned above, most findings about this gene are about the promotion of human neocortex amplification and basal progenitor development [10, 84–86]. However, the molecular mechanism of this gene in the BC process has not been identified, but it is well documented. Unlike *ARHGAP11A*, *ARHGAP11B* does not show RhoGAP activity thanks to a lack of the RhoGAP domain [35, 36]. To find possible roles and pathways for *ARHGAP11B* and mainly *ARHGAP11B*, we investigated coexpressed genes with these two genes using LinkedOmics. We found out that not only did both genes have similar shared positive and negative expression correlations with other genes, but they also showed a positive correlation expression with each other, indicating the involvement of common pathways for them. Moreover, we looked for GSEA using LinkedOmics which revealed some similar BP terms and KEGG pathways for them, representing similar roles for *ARHGAP11B* in BLBC malignancy through the ER or HER2 receptor signaling pathways due to its expression correlation with *ARHGAP11A*. However, it needs more research to uncover the exact molecular mechanisms of *ARHGAP11B* in BC.

As mentioned above, our methylation analysis showed that the promoter methylation of *ARHGAP11A* and *ARHGAP11B* had a significant negative correlation with their upregulation (Figure 1). Indeed, the promoter hypomethylation of these genes can result in their mRNA upregulation. It can be concluded that the promoters of these genes are hypomethylated in IDC tumors, leading to their overexpression in this subtype of BC. Also, we found out that these genes had higher expression in all N0, N1, N2, and N3 (especially in N2) compared to the matched normal tissues; therefore, in addition to their roles in tumorigenesis and invasion, they might have roles in the LNM process. Moreover, based on TCGA cancer data given in Results, ARHGAP11A and ARHGAP11B are highly overexpressed in cervical cancers, another area where estrogen and progesterone play important roles. Therefore, it is also proposed to investigate these genes and their function in cervical cancer to confirm the possible common pathways in the cancers with the role of estrogen and progesterone.

## 5. Conclusion

Totally, the following results from our study can ultimately propose that these paralog genes can have protooncogenic roles in the process of malignancy and pathogenicity of IDC as a major invasive type of BC: (1) the overexpression of *ARHGAP11A* and *ARHGAP11B* tumor samples versus their adjacent normal tissues in IDC cases. (2) Their overexpression in different reproductive and clinicopathological factors such as lack or short-term breastfeeding, early age at menarche, and regular menstrual cycles for *ARHGAP11A*, and in ER and HER2 positive tumors for *ARHGAP11B* in women with IDC. (3) Using TCGA-BRCA data analysis, we showed the possible oncogenic role of these genes, especially in TNBCs, and also, based on histological analysis, these genes were specific to the IDC subtype. Furthermore, they showed a positive correlation between several genes and also each other in TCGA-BRCA. Moreover, TCGA-BRCA analysis revealed their possible roles in tumorigenesis, invasion, and nodal metastasis in breast tumors. Thus, these may be proposed as biomarkers of all BRCA subclasses, mainly triple-negative ones. These genes can be considered possible diagnostic, prognostic, and therapeutic biomarkers for IDC. However, this research needs more investigations in the future to identify more details about the molecular mechanisms of BC, and future studies may suggest these two genes as candidates for tumor biomarkers in combination with biomarker panels in different types of cancer, especially various types of BC.

## Figures and Tables

**Table 1:** Characteristics of the population study and the results of *ARHGAP11A* and *ARHGAP11B* gene expression levels in the variable subgroups.

Variables	Subgroups	*N*	*ARHGAP11AA*			*ARHGAP11B*		
			MEAN	SD	*P* value	MEAN	SD	*P* value
Age	<40	15	9.749	12.377	0.901	7.864	10.489	0.444
	≥40	36	7.440	8.771		9.903	10.568	

Marital status	Married	43	8.328	9.714	0.422	9.704	10.318	0.147
	Single	8	6.996	11.451		7.152	11.820	

Age at first marriage	<18	16	10.626	10.487	0.157	13.593	13.507	0.492
	18-24	19	5.2723	6.714		8.364	7.936	
	≥24	9	10.795	12.179		6.432	5.938	

Parity	Parous	36	8.898	10.151	0.264	10.64	10.898	0.131
	Nulliparous	15	6.250	9.307		6.075	8.923	

Age at menarche (year)	<14	36	9.014	9.597	**0.041**	10.572	11.769	0.508
	≥14	15	5.971	10.597		6.259	5.656	

Menstrual cycles	Regular	43	16.266	13.850	**0.013**	7.969	9.338	0.062
	Irregular	8	6.603	8.340		16.477	13.846	

Menopausal status	Pre	4	10.491	17.916	0.551	4.870	2.354	0.889
	Post	47	7.917	9.215		9.681	10.826	

Age at FFTP (year)	<25	38	8.164	9.682	0.800	10.505	11.458	0.310
	≥25	10	9.836	11.881		5.543	6.086	

Abortion history	Yes	12	6.616	10.26	0.609	7.519	9.612	0.500
	No	37	8.917	10.01		9.952	10.98	

Breastfeeding experiences	Yes	37	8.681	10.096	0.423	10.415	10.839	0.170
	No	14	6.633	9.534		6.365	9.185	

Breastfeeding (total months)	0-6	20	11.605	11.729	**0.034**	6.864	8.463	0.467
	6-24	11	7.3245	7.365		11.000	11.674	
	≥24	20	5.0713	8.277		10.651	11.601	

OCP consumption	Yes	10	10.409	11.685	0.420	14.776	13.107	0.068
	No	41	7.561	9.4864		7.969	9.453	

Abbreviations: FFTP: first full-term pregnancy; OCP: oral contraceptive pills; *N*: number of participants. Bold values are statistically significant (*P* < 0.05).

**Table 2:** *ARHGAP11A* expression in TCGA cancers.

Cancer type	Fold change	log2 (fold change)	Mean TPM (tumor)	Mean TPM (normal)	*P* value	Adjusted *P* value
Breast invasive carcinoma	4.69	2.23	480.03	102.32	3.77*e*‐50	7.52*e*‐49
Pan-kidney cohort (KICH+KIRC+KIRP)	2.95	1.56	135.41	45.88	1.36*e*‐47	2.28*e*‐46
Lung squamous cell carcinoma	8.44	3.08	766.07	90.72	1.96*e*‐31	1.3*e*‐29
Kidney renal clear cell carcinoma	2.74	1.45	160.72	58.66	1.78*e*‐30	1.49*e*‐29
Lung adenocarcinoma	4.35	2.12	394.06	90.63	1.77*e*‐27	2.56*e*‐26
Stomach and esophageal carcinoma	4.08	2.03	1000.62	245.54	1.53*e*‐23	4.71*e*‐21
Liver hepatocellular carcinoma	6.27	2.65	235.47	37.56	1.91*e*‐23	7.06*e*‐22
Uterine corpus endometrial carcinoma	6.59	2.72	511.13	77.58	2.15*e*‐20	8.37*e*‐19
Stomach adenocarcinoma	3.78	1.92	922.81	244.37	2.99*e*‐17	5.44*e*‐15
Head and neck squamous cell carcinoma	2.24	1.16	760.27	339.19	1.39*e*‐14	2.1*e*‐13
Kidney renal papillary cell carcinoma	2.7	1.43	84.99	31.48	1.21*e*‐12	8.74*e*‐12
Colon adenocarcinoma	1.89	0.92	770.75	406.89	8.73*e*‐11	2.47*e*‐10
Kidney chromophobe	5.51	2.46	160.32	29.09	1.58*e*‐10	1.34*e*‐9
Bladder urothelial carcinoma	2.55	1.35	579.21	227.08	2.56*e*‐8	3.52*e*‐7
Esophageal carcinoma	4.71	2.23	1174.79	249.58	2.92*e*‐7	2.7*e*‐05
Prostate adenocarcinoma	1.64	0.71	95.69	58.33	3.5*e*‐7	1.1*e*‐06
Cholangiocarcinoma	4.78	2.26	273.22	57.11	1*e*‐05	5.3*e*‐05
Cervical squamous cell carcinoma and endocervical adenocarcinoma	29.52	4.88	936.65	31.73	0.0029	0.0418
Thyroid carcinoma	1.14	0.19	84.17	73.93	0.0035	0.00518
Rectum adenocarcinoma	1.47	0.56	722.45	491.06	0.0352	0.0595

TMP: Transcript per million.

**Table 3:** *ARHGAP11B* expression in TCGA cancers.

Cancer type	Fold change	log2 (fold change)	Mean TPM (tumor)	Mean TPM (normal)	*P* value	Adjusted *P* value
Breast invasive carcinoma	4.69	2.23	480.03	102.32	3.77*e*‐50	7.52*e*‐49
Pan-kidney cohort (KICH+KIRC+KIRP)	2.95	1.56	135.41	45.88	1.36*e*‐47	2.28*e*‐46
Lung squamous cell carcinoma	8.44	3.08	766.07	90.72	1.96*e*‐31	1.3*e*‐29
Kidney renal clear cell carcinoma	2.74	1.45	160.72	58.66	1.78*e*‐30	1.49*e*‐29
Lung adenocarcinoma	4.35	2.12	394.06	90.63	1.77*e*‐27	2.56*e*‐26
Stomach and esophageal carcinoma	4.08	2.03	1000.62	245.54	1.53*e*‐23	4.71*e*‐21
Liver hepatocellular carcinoma	6.27	2.65	235.47	37.56	1.91*e*‐23	7.06*e*‐22
Uterine corpus endometrial carcinoma	6.59	2.72	511.13	77.58	2.15*e*‐20	8.37*e*‐19
Stomach adenocarcinoma	3.78	1.92	922.81	244.37	2.99*e*‐17	5.44*e*‐15
Head and neck squamous cell carcinoma	2.24	1.16	760.27	339.19	1.39*e*‐14	2.1*e*‐13
Kidney renal papillary cell carcinoma	2.7	1.43	84.99	31.48	1.21*e*‐12	8.74*e*‐12
Colon adenocarcinoma	1.89	0.92	770.75	406.89	8.73*e*‐11	2.47*e*‐10
Kidney chromophobe	5.51	2.46	160.32	29.09	1.58*e*‐10	1.34*e*‐9
Bladder urothelial carcinoma	2.55	1.35	579.21	227.08	2.56*e*‐8	3.52*e*‐7
Esophageal carcinoma	4.71	2.23	1174.79	249.58	2.92*e*‐7	2.7*e*‐05
Prostate adenocarcinoma	1.64	0.71	95.69	58.33	3.5*e*‐7	1.1*e*‐06
Cholangiocarcinoma	4.78	2.26	273.22	57.11	1*e*‐05	5.3*e*‐05
Cervical squamous cell carcinoma and endocervical adenocarcinoma	29.52	4.88	936.65	31.73	0.0029	0.0418
Thyroid carcinoma	1.14	0.19	84.17	73.93	0.0035	0.00518
Rectum adenocarcinoma	1.47	0.56	722.45	491.06	0.0352	0.0595

TMP: Transcript per million.

**Table 4:** Pathological data of the tumor samples from BC patients.

Variables	Subgroups	N	*ARHGAP11A*			*ARHGAP11B*		
			MEAN	SD	*P* values	MEAN	SD	*P* values
ER	Pos	45	8.401	10.101	0.604	10.028	10.864	**0.041**
	Neg	6	6.001	4.448		3.873	4.448	

PR	Pos	34	8.124	9.759	0.920	10.439	11.697	0.484
	Neg	17	8.109	10.460		7.034	7.274	

HER2	Pos	21	10.563	10.720	0.063	10.550	12.278	**0.031**
	Neg	30	6.408	9.063		8.431	9.139	

Abbreviations: Pos: positive; Neg: negative; *N*: number of participants. Bold values are statistically significant (*P* < 0.05).

## Data Availability

All data used to support the findings of this study are included in the article.

## Supplementary Materials

Supplementary 1 File S1: correlation between RNA expression and methylation of CpG island positions in *ARHGAP11A* and *B*.

Supplementary 2 File S2: BP, MF, CC, and KEGG pathways involved in negatively (orange) and positively (blue) coexpressed genes with *ARHGAP11A* in TCGA-BRCA.

Supplementary 3 File S3: BP and KEGG pathways involved in negatively (orange) and positively coexpressed (blue) genes with *ARHGAP11B* in TCGA-BRCA.

Supplementary 4 File S4: specific GSEA in terms of BP and KEGG pathways for coexpressed genes to *ARHGAP11A* and *ARHGAP11B* and also their common GSEA.

Supplementary 5 File S5: *ARHGAP11A* and *B* expression from TCGA-BRCA in different races (Caucasian, African-American, and Asian).

Supplementary 6 File S6: correlation between expression of ARHGAP11A and OS possibility in different types of breast cancer.

Supplementary 7 File S7: correlation between expression of ARHGAP11B and OS possibility in different types of breast cancer.
