# Supplementary material for: Protooncogenic Role of ARHGAP11A and ARHGAP11B in Invasive Ductal Carcinoma: Two Promising Breast Cancer Biomarkers
Source: Biomed Res Int. 2023 Nov 23;2023:8236853. doi: 10.1155/2023/8236853 (PMC10689071; doi:10.1155/2023/8236853)
Supplement: Supplementary 1 — File S1: correlation between RNA expression and methylation of CpG island positions in ARHGAP11A and B. [file 8236853.f1.pdf]

a-1) ARHGAP11A Isoform expression in TCGA-BRCA

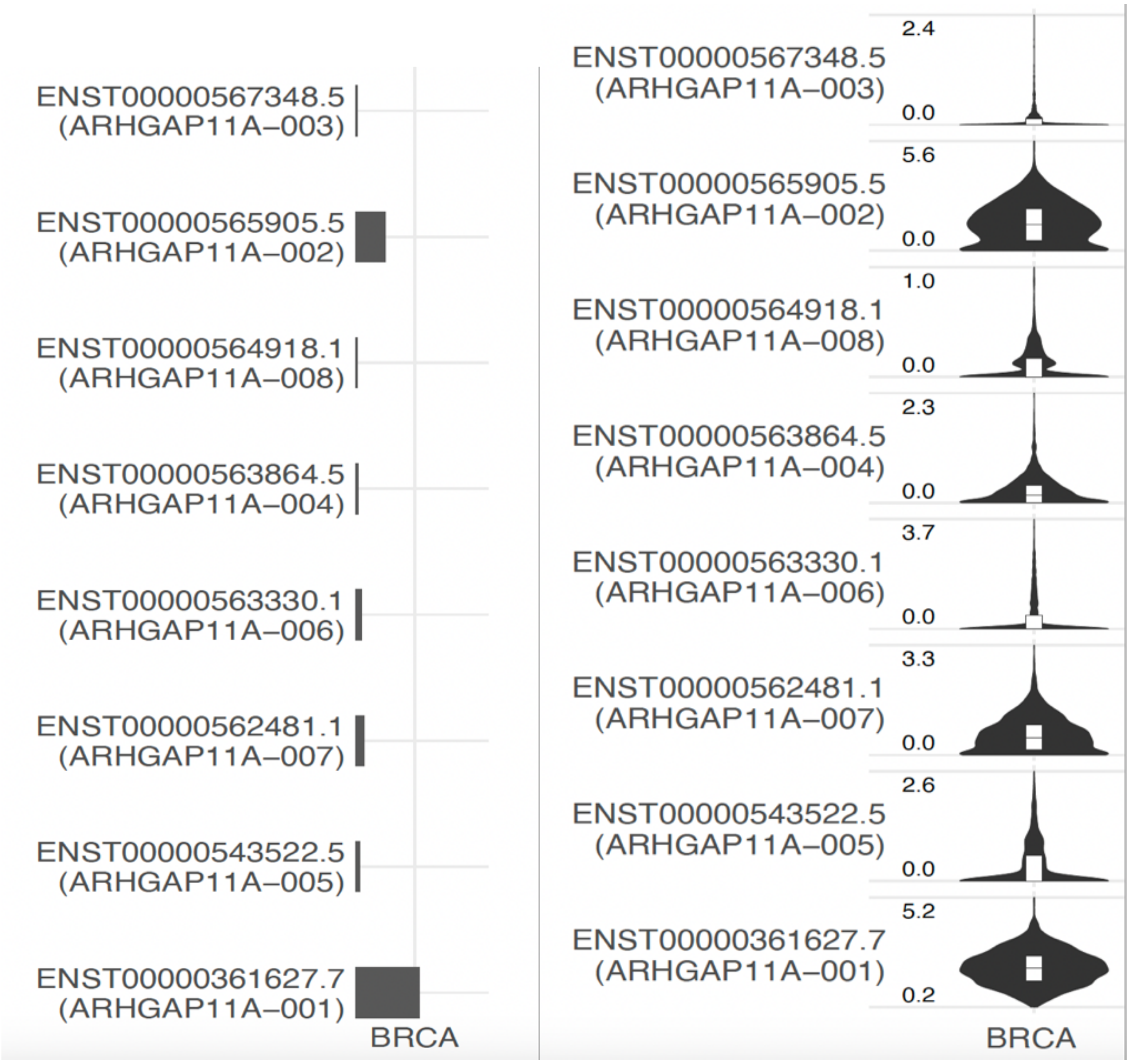

a-2) The correlation between the ARHGAP11A expression (ENST00000361627.7 isoform) (transcript-level) and DNA methylation in all individual CpGs

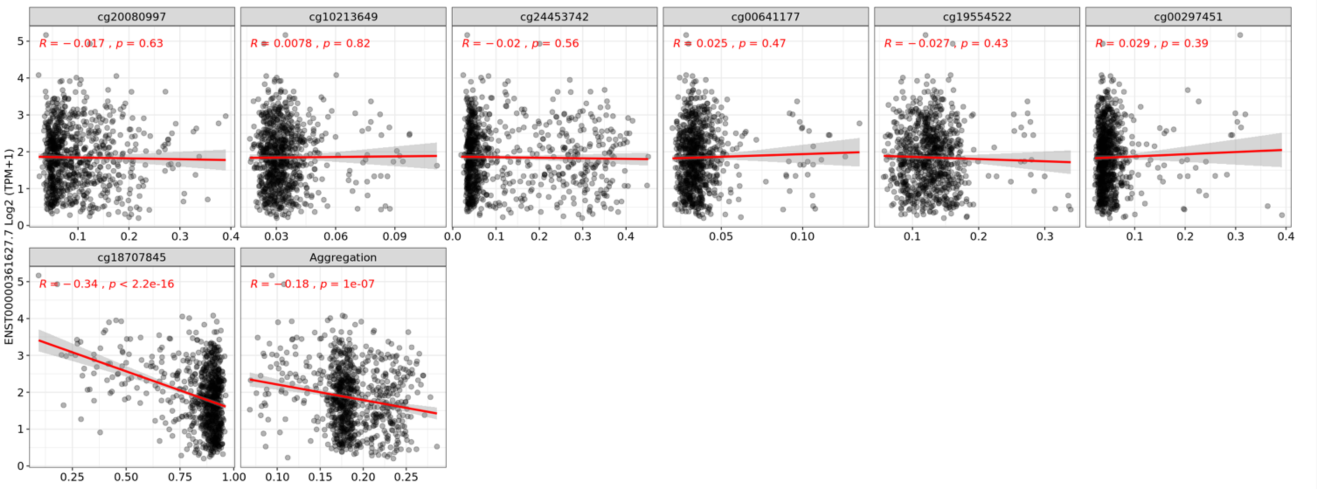

a-3) Probes used for the individual CpGs of ARHGAP11A

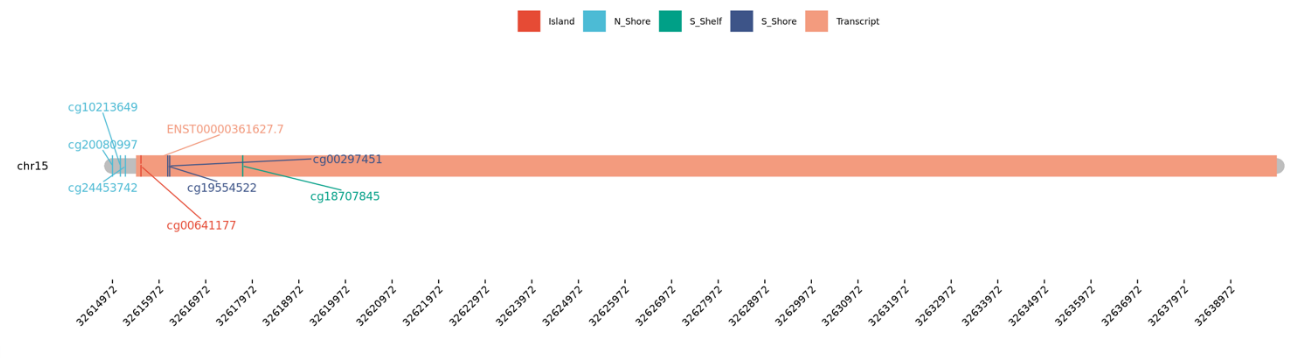

Probe	CGIposition	Distance_To_TSS
cg20080997	N_Shore	518
cg10213649	N_Shore	349
cg24453742	N_Shore	241
cg00641177	Island	-93
cg19554522	S_Shore	-668
cg00297451	S_Shore	-709
cg18707845	S_Shelf	-2277

b-1) ARHGAP11B Isoform expression in TCGA-BRCA

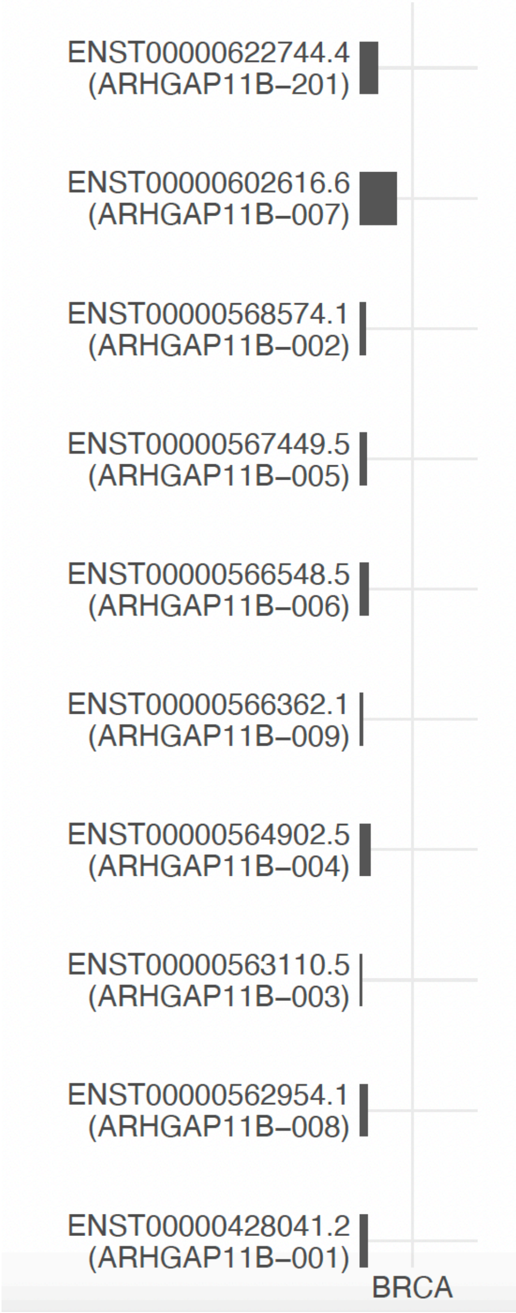

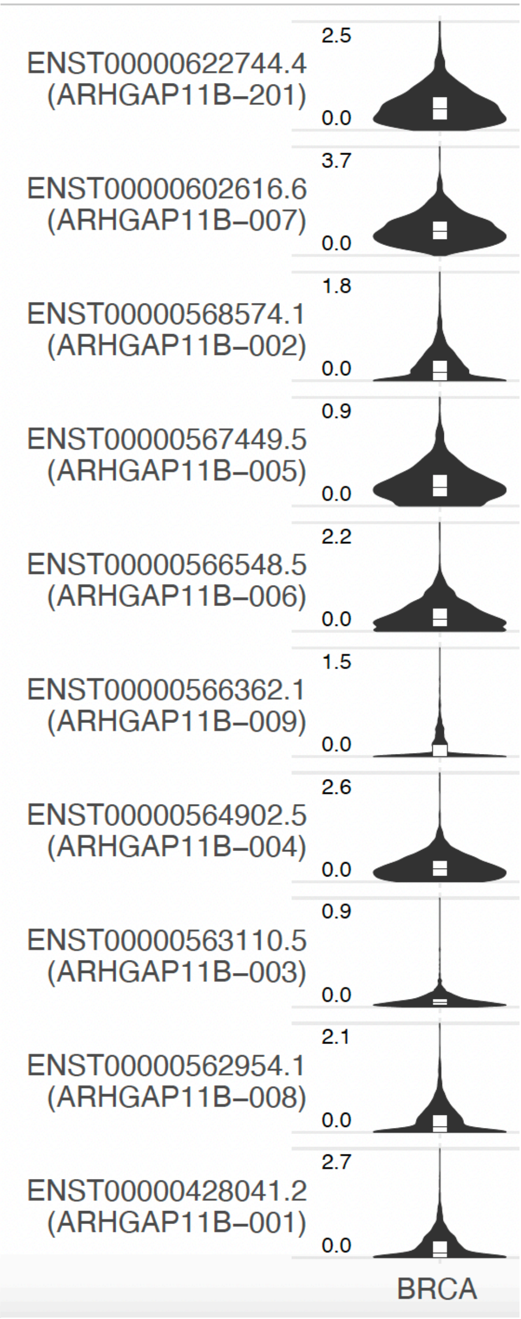

b-2) The correlation between the ARHGAP11B expression (ENST00000602616.6 isoform) (transcript-level) and DNA methylation in all individual CpGs

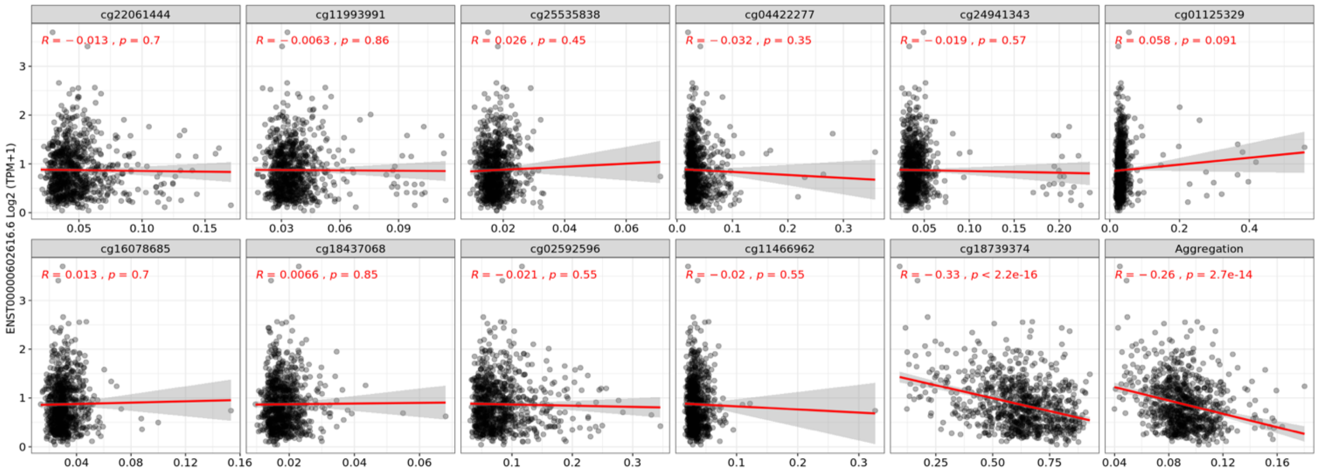

B-3) Probes used for the individual CpGs of ARHGAP11B

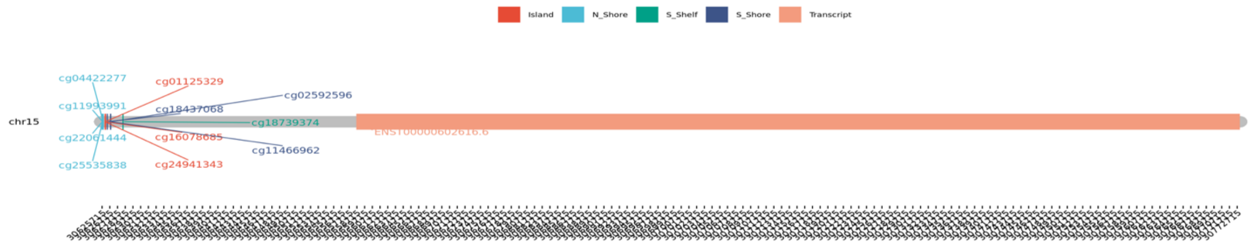

Probe	CGIposition	Distance_To_TSS
cg22061444	N_Shore	33002
cg11993991	N_Shore	32962
cg25535838	N_Shore	32929
cg04422277	N_Shore	32855
cg24941343	Island	32607
cg01125329	Island	32592
cg16078685	Island	32581
cg18437068	S_Shore	32325
cg02592596	S_Shore	31906
cg11466962	S_Shore	31900
cg18739374	S_Shelf	30293
