# Supplementary material for: Protooncogenic Role of ARHGAP11A and ARHGAP11B in Invasive Ductal Carcinoma: Two Promising Breast Cancer Biomarkers
Source: Biomed Res Int. 2023 Nov 23;2023:8236853. doi: 10.1155/2023/8236853 (PMC10689071; doi:10.1155/2023/8236853)
Supplement: Supplementary 2 — File S2: BP, MF, CC, and KEGG pathways involved in negatively (orange) and positively (blue) coexpressed genes with ARHGAP11A in TCGA-BRCA. [file 8236853.f2.pdf]

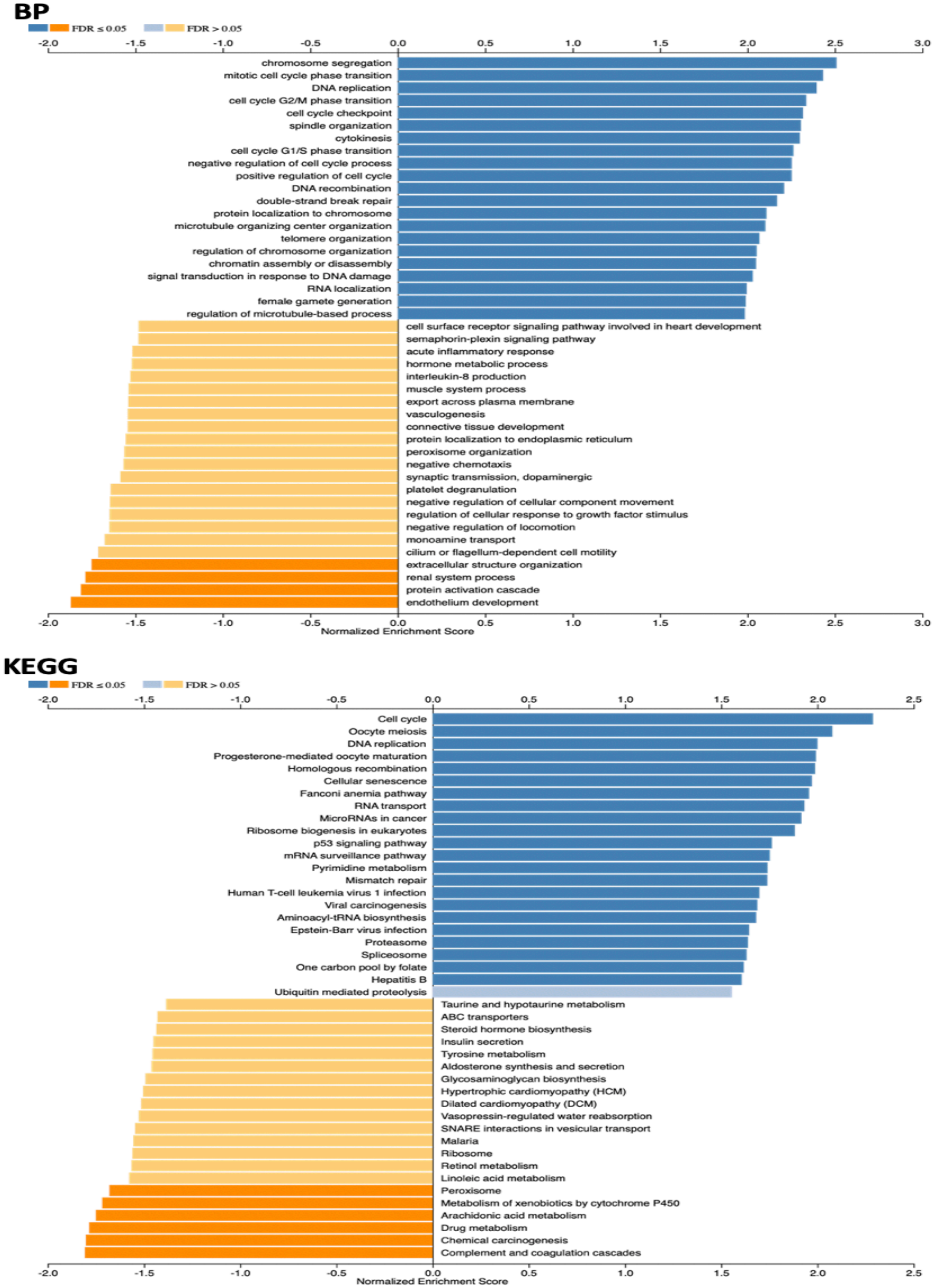

Supplementary File S2. BP, MF, CC, KEGG pathways involved in negatively (orange) and positively (blue) co-expressed genes with ARHGAP11A in TCGA-BRCA, respectively.
