# Supplementary material for: Protooncogenic Role of ARHGAP11A and ARHGAP11B in Invasive Ductal Carcinoma: Two Promising Breast Cancer Biomarkers
Source: Biomed Res Int. 2023 Nov 23;2023:8236853. doi: 10.1155/2023/8236853 (PMC10689071; doi:10.1155/2023/8236853)
Supplement: Supplementary 3 — File S3: BP and KEGG pathways involved in negatively (orange) and positively coexpressed (blue) genes with ARHGAP11B in TCGA-BRCA. [file 8236853.f3.pdf]

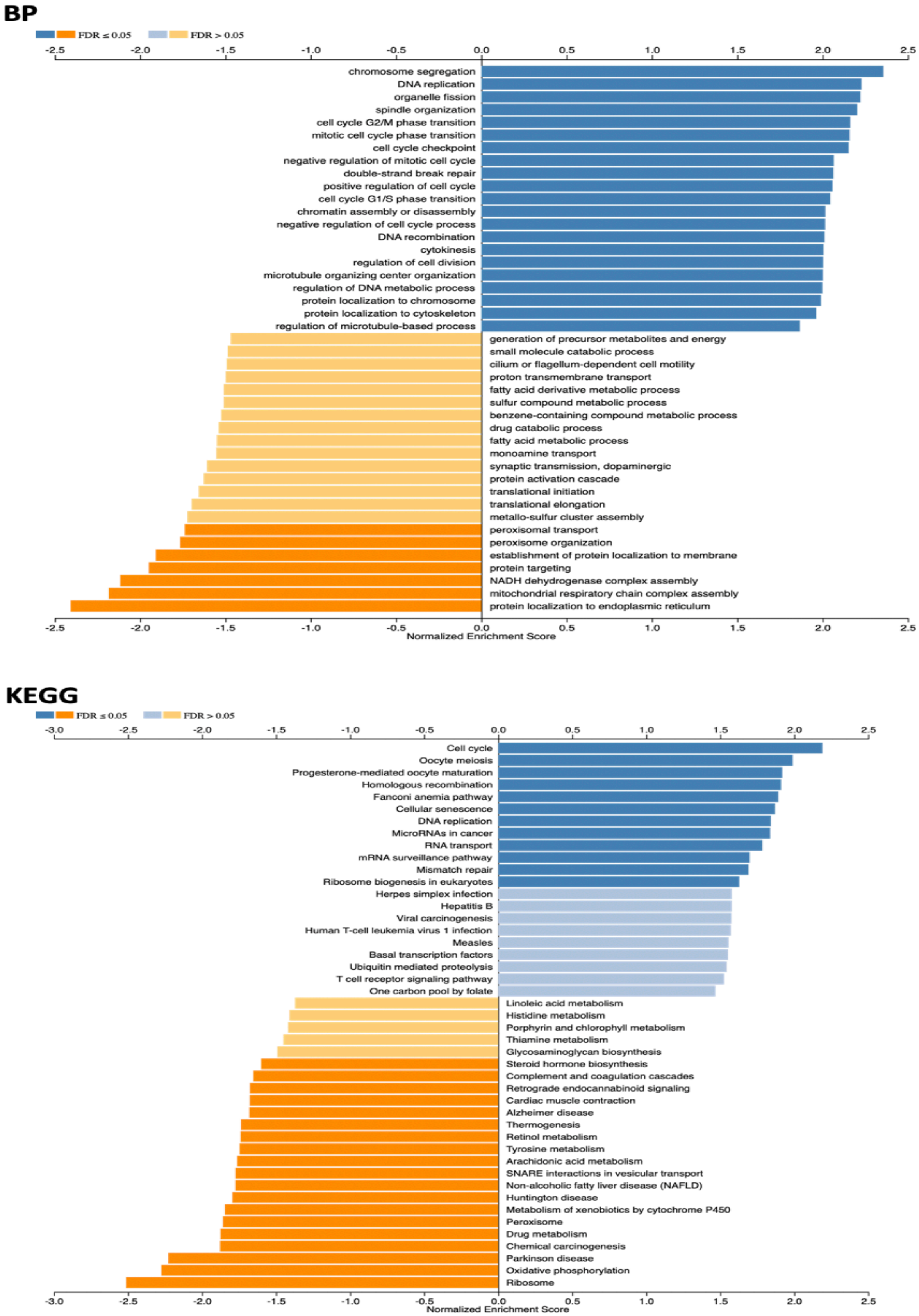


**Supplementary File S3** BP and KEGG pathways involved in negatively (orange) and positively co-expressed (blue) genes with *ARHGAP11B* in TCGA-BRCA, respectively.
