# Supplementary material for: Protooncogenic Role of ARHGAP11A and ARHGAP11B in Invasive Ductal Carcinoma: Two Promising Breast Cancer Biomarkers
Source: Biomed Res Int. 2023 Nov 23;2023:8236853. doi: 10.1155/2023/8236853 (PMC10689071; doi:10.1155/2023/8236853)
Supplement: Supplementary 4 — File S4: specific GSEA in terms of BP and KEGG pathways for coexpressed genes to ARHGAP11A and ARHGAP11B and also their common GSEA. [file 8236853.f4.pdf]

Table 1. GSEA: BPs related to positively co-expressed genes to ARHGAP11A and ARHGAP11B

**BPs related to positively correlated genes found only in ARHGAP11A**	**Common BPs related to positively correlated genes found in both ARHGAP11A and ARHGAP11B**	**BPs related to positively correlated genes found only in ARHGAP11B**
Telomer organization	Chromosome segregation	Organelle fission
Regulation of chromosome organization	DNA replication	Negative regulation of mitotic cell cycle
Signal transduction in response to DNA damage	Spindle organization	Regulation of cell division
RNA localization	Cell cycle G2/M phase transition	Regulation of DNA metabolic process
Female gamete generation	Mitotic cell cycle phase transition	Protein localization to cytoskeleton
	Cell cycle checkpoint	
	Double-strand break repair	
	Positive regulation of cell cycle	
	Cell cycle G1/S phase transition	
	Chromatin assembly or disassembly	
	Negative regulation of cell cycle process	
	DNA recombination	
	cytokinesis	
	Microtubule organizing center organization	
	Protein localization to chromosome	
	Regulation of microtubule-based process	

Table 2. GSEA: BPs related to negatively co-expressed genes to ARHGAP11A and ARHGAP11B

**BPs related to negatively correlated genes found only in ARHGAP11A**	**BPs related to negatively correlated genes found in both ARHGAP11A and ARHGAP11B**	**BPs related to negatively correlated genes found only in ARHGAP11B**
		Protein localization to endoplasmic reticulum
		Peroxisome organization
		Peroxisomal transport
		Establishment of protein localization to membrane
Protein activation cascade		Protein targeting
Extracellular structure organization		NADH dehydrogenase complex assembly

Renal system process		Mitochondrial respiratory chain complex assembly
Endothelium development		

Table 3. GSEA: KEGG pathways related to positively co-expressed genes to ARHGAP11A and ARHGAP11B

**KEGGs related to positively correlated genes found only in ARHGAP11A**	**Common KEGGs related to positively correlated genes found in both ARHGAP11A and ARHGAP11B**	**KEGGs related to positively correlated genes found only in ARHGAP11B**
P53 signaling pathway	Cell cycle	
Pyrimidine metabolism	Oocyte meiosis	
Aminoacytyl-tRNA biosynthesis	Progesterone-mediated oocyte maturation	
Epstein-Barr virus infection	Homologous recombination	
Proteasome	Fanconi anemia pathway	
Spliceosome	Cellular senescence	
Hepatitis B	DNA replication	
Viral carcinogenesis	microRNAs in cancer	
Human T-cell leukemia virus 1 infection	RNA transport	
One carbon pool by folate	mRNA surveillance pathway	
	Mismatch repair	
	Ribosome biogenesis in eukaryotes	

Table 4. GSEA: KEGG pathways related to negatively co-expressed genes to ARHGAP11A and ARHGAP11B

**KEGGs related to negatively correlated genes found only in ARHGAP11A**	**Common KEGGs related to negatively correlated genes found in both ARHGAP11A and ARHGAP11B**	**KEGGs related to negatively correlated genes found only in ARHGAP11B**
	Complement and coagulation cascades	Steroid hormone biosynthesis
	Arachidonic acid metabolism	Retinol metabolism
	Metabolism of xenobiotics by cytochrome P450	Tyrosine metabolism
	Peroxisome	Retrograde endocannabinoid signaling
	Drug metabolism	Cardiac muscle contraction
	Chemical carcinogenesis	Alzheimer disease
		Thermogenesis
		SNARE interaction in vesicular transport
		Non-alcoholic fatty liver disease (NAFLD)

		Huntington disease
		Parkinson disease
		Oxidative phosphorylation
		ribosome
