# Supplementary material for: Protooncogenic Role of ARHGAP11A and ARHGAP11B in Invasive Ductal Carcinoma: Two Promising Breast Cancer Biomarkers
Source: Biomed Res Int. 2023 Nov 23;2023:8236853. doi: 10.1155/2023/8236853 (PMC10689071; doi:10.1155/2023/8236853)
Supplement: Supplementary 5 — File S5: ARHGAP11A and B expression from TCGA-BRCA in different races (Caucasian, African-American, and Asian). [file 8236853.f5.pdf]

a

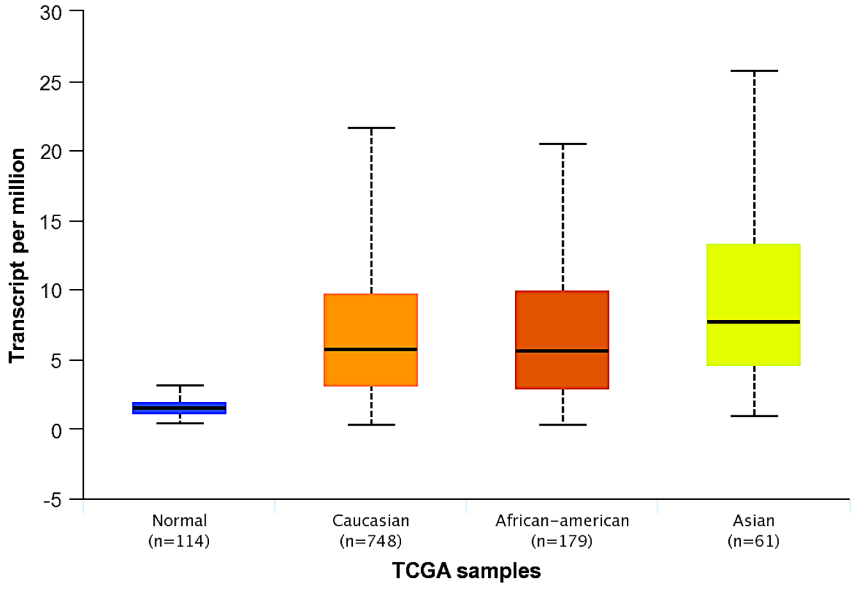

Comparison	Statistical significance
Normal-vs-Caucasian	1.6E-12
Normal-vs-AfricanAmerican	1.6E-12
Normal-vs-Asian	1.3E-07
Caucasian-vs-AfricanAmerican	8.2E-01
Caucasian-vs-Asian	6.6E-02
AfricanAmerican-vs-Asian	6.2E-02

b

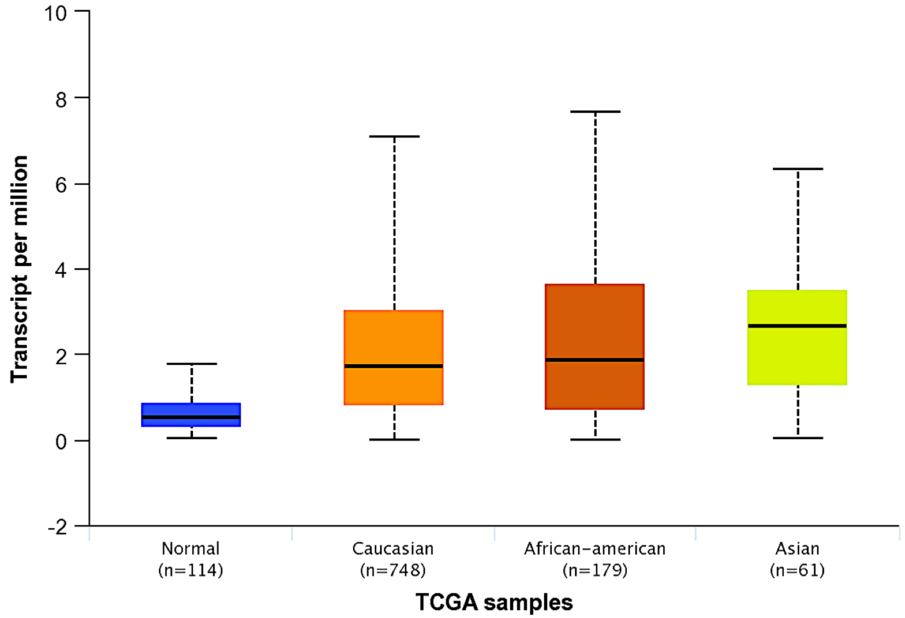

Comparison	Statistical significance
Normal-vs-Caucasian	<1E-12
Normal-vs-AfricanAmerican	1.6E-12
Normal-vs-Asian	3.5E-05
Caucasian-vs-AfricanAmerican	7.3E-01
Caucasian-vs-Asian	2. 9E-01
AfricanAmerican-vs-Asian	3.8E-01
