# Supplementary material for: Protooncogenic Role of ARHGAP11A and ARHGAP11B in Invasive Ductal Carcinoma: Two Promising Breast Cancer Biomarkers
Source: Biomed Res Int. 2023 Nov 23;2023:8236853. doi: 10.1155/2023/8236853 (PMC10689071; doi:10.1155/2023/8236853)

**RNAseq ID:**	ARHGAP11A
**Survival:**	OS
**Auto select best cutoff:**	checked
**Follow up threshold:**	all
**Censore at threshold:**	checked
**Compute median over entire database:**	false
**Cutoff value used in analysis:**	-0.84
**Expression range of the probe:**	-3 - 2
**Probe set option:**	user selected probe set
**Invert HR values below 1:**	not checked

Restrictions

Lymph node status:	all
ER status:	all
PGR status:	all
HER2 status:	all
KI67 status:	all
Nottingham histologic grade:	all
PAM50 subtype:	all

Cohorts

Endocrine treated:	all
Chemo treated:	all

Results

P value: 0.0012
FDR: over 50%

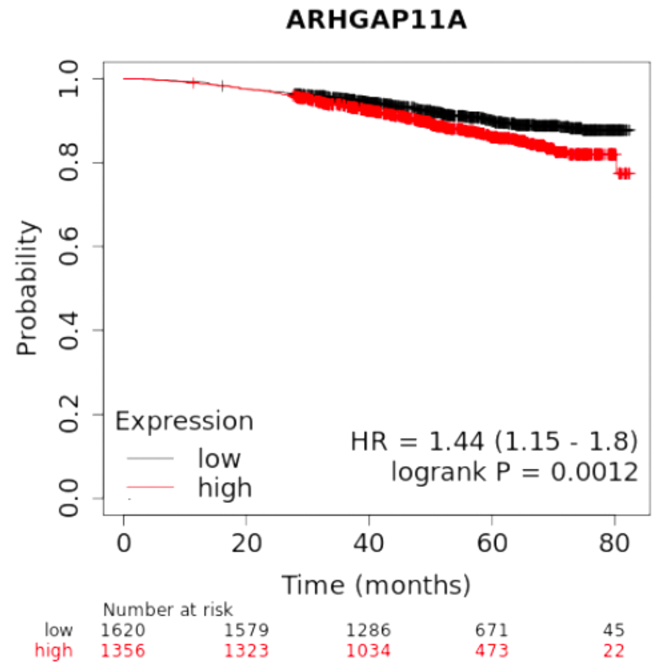

**RNAseq ID:**	ARHGAP11A -
**Survival:**	OS
**Auto select best cutoff:**	checked
**Follow up threshold:**	all
**Censore at threshold:**	checked
**Compute median over entire database:**	false
**Cutoff value used in analysis:**	-1.08
**Expression range of the probe:**	-3 - 1
**Probe set option:**	user selected probe set
**Invert HR values below 1:**	not checked

Restrictions

Lymph node status:	all
ER status:	all
PGR status:	all
HER2 status:	all
KI67 status:	all
Nottingham histologic grade:	all
PAM50 subtype:	Normal

Cohorts

Endocrine treated:	all
Chemo treated:	all

Results

P value: 0.4958
FDR: 100%

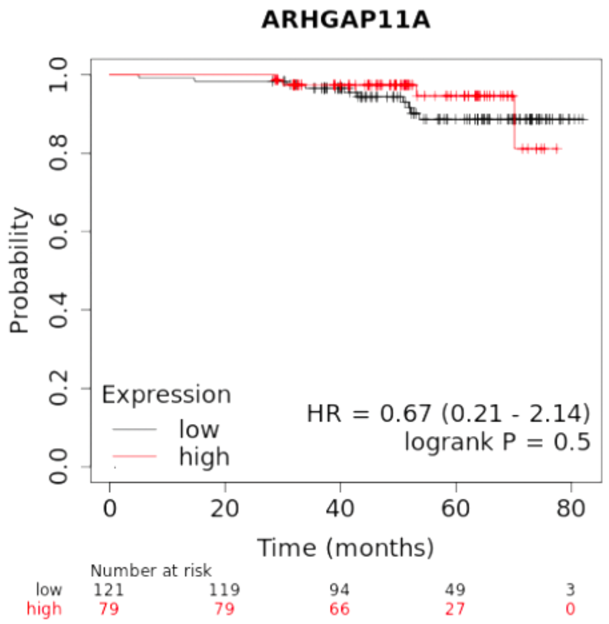

**RNAseq ID:**	ARHGAP11A
**Survival:**	OS
**Auto select best cutoff:**	checked
**Follow up threshold:**	all
**Censore at threshold:**	checked
**Compute median over entire database:**	false
**Cutoff value used in analysis:**	-0.24
**Expression range of the probe:**	-3 - 2
**Probe set option:**	user selected probe set
**Invert HR values below 1:**	not checked

Restrictions

Lymph node status:	all
ER status:	all
PGR status:	all
HER2 status:	all
KI67 status:	all
Nottingham histologic grade:	all
PAM50 subtype:	Basal

Cohorts

Endocrine treated:	all
Chemo treated:	all

Results

P value: 0.2448
FDR: 100%

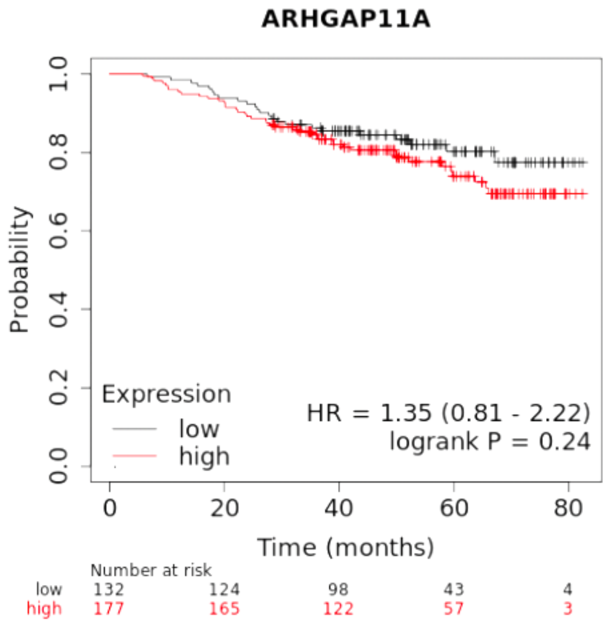

**RNAseq ID:**	ARHGAP11A
**Survival:**	OS
**Auto select best cutoff:**	checked
**Follow up threshold:**	all
**Censore at threshold:**	checked
**Compute median over entire database:**	false
**Cutoff value used in analysis:**	-1.65
**Expression range of the probe:**	-3 - 1
**Probe set option:**	user selected probe set
**Invert HR values below 1:**	not checked

Restrictions

Lymph node status:	all
ER status:	all
PGR status:	all
HER2 status:	all
KI67 status:	all
Nottingham histologic grade:	all
PAM50 subtype:	LuminalA

Cohorts

Endocrine treated:	all
Chemo treated:	all

Results

P value: 0.3061
FDR: 100%

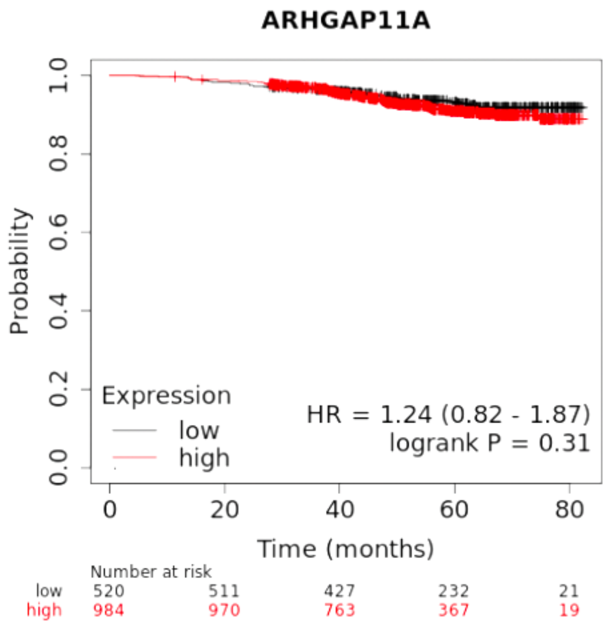

**RNAseq ID:**	ARHGAP11A
**Survival:**	OS
**Auto select best cutoff:**	checked
**Follow up threshold:**	all
**Censore at threshold:**	checked
**Compute median over entire database:**	false
**Cutoff value used in analysis:**	-0.58
**Expression range of the probe:**	-3 - 2
**Probe set option:**	user selected probe set
**Invert HR values below 1:**	not checked

Restrictions

Lymph node status:	all
ER status:	all
PGR status:	all
HER2 status:	all
KI67 status:	all
Nottingham histologic grade:	all
PAM50 subtype:	LuminalB

Cohorts

Endocrine treated:	all
Chemo treated:	all

Results

P value: 0.039
FDR: over 50%

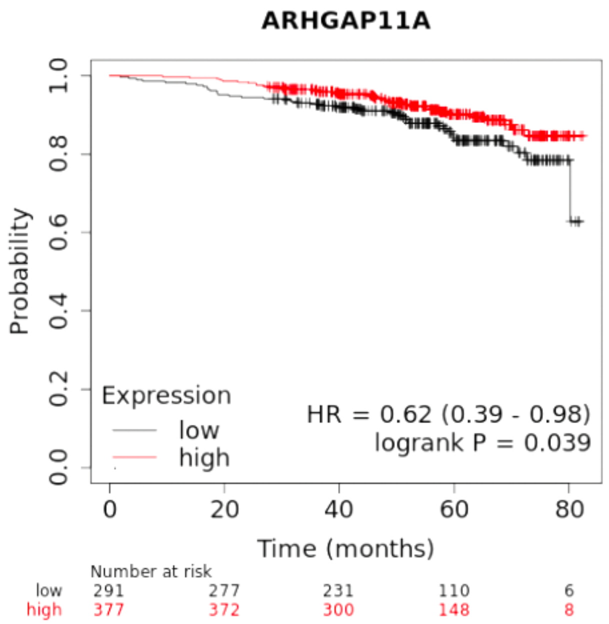

**RNAseq ID:**	ARHGAP11A -
**Survival:**	OS
**Auto select best cutoff:**	checked
**Follow up threshold:**	all
**Censore at threshold:**	checked
**Compute median over entire database:**	false
**Cutoff value used in analysis:**	-1.19
**Expression range of the probe:**	-3 - 1
**Probe set option:**	user selected probe set
**Invert HR values below 1:**	not checked

Restrictions

Lymph node status:	all
ER status:	all
PGR status:	all
HER2 status:	all
KI67 status:	all
Nottingham histologic grade:	all
PAM50 subtype:	Her2

Cohorts

Endocrine treated:	all
Chemo treated:	all

Results

P value: 0.1323
FDR: 100%

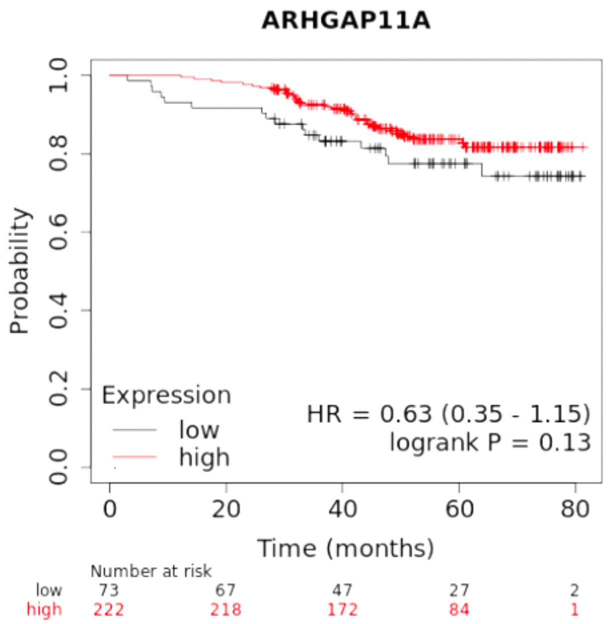

Supplement: Supplementary 6 — File S6: correlation between expression of ARHGAP11A and OS possibility in different types of breast cancer. [file 8236853.f6.pdf]
