# Supplementary material for: Protooncogenic Role of ARHGAP11A and ARHGAP11B in Invasive Ductal Carcinoma: Two Promising Breast Cancer Biomarkers
Source: Biomed Res Int. 2023 Nov 23;2023:8236853. doi: 10.1155/2023/8236853 (PMC10689071; doi:10.1155/2023/8236853)

**RNAseq ID:**	ARHGAP11B
**Survival:**	OS
**Auto select best cutoff:**	checked
**Follow up threshold:**	all
**Censore at threshold:**	checked
**Compute median over entire database:**	false
**Cutoff value used in analysis:**	2.86
**Expression range of the probe:**	-1 - 5
**Probe set option:**	user selected probe set
**Invert HR values below 1:**	not checked

Restrictions

Lymph node status:	all
ER status:	all
PGR status:	all
HER2 status:	all
KI67 status:	all
Nottingham histologic grade:	all
PAM50 subtype:	all

Cohorts

Endocrine treated:	all
Chemo treated:	all

Results

P value: 0.0012
FDR: over 50%

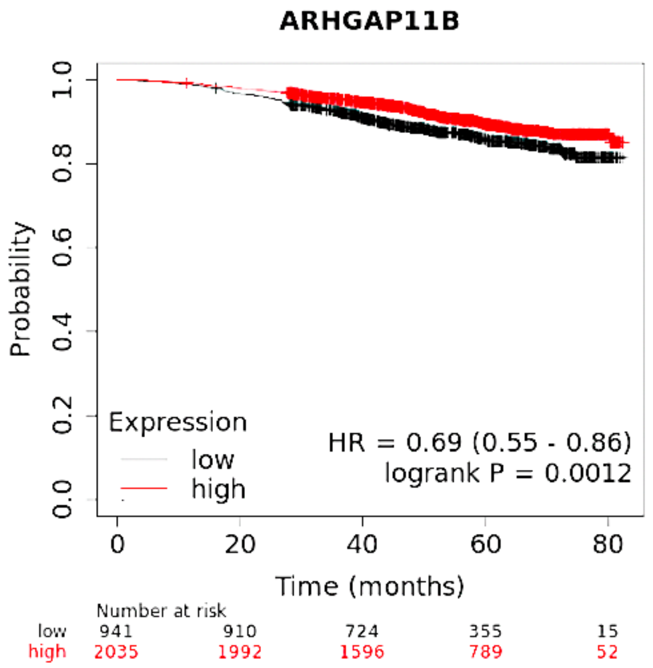

**RNAseq ID:**	ARHGAP11B
**Survival:**	OS
**Auto select best cutoff:**	checked
**Follow up threshold:**	all
**Censore at threshold:**	checked
**Compute median over entire database:**	false
**Cutoff value used in analysis:**	3.17
**Expression range of the probe:**	2 - 5
**Probe set option:**	user selected probe set
**Invert HR values below 1:**	not checked

Restrictions

Lymph node status:	all
ER status:	all
PGR status:	all
HER2 status:	all
KI67 status:	all
Nottingham histologic grade:	all
PAM50 subtype:	Normal

Cohorts

Endocrine treated:	all
Chemo treated:	all

Results

P value: 0.3658
FDR: 100%

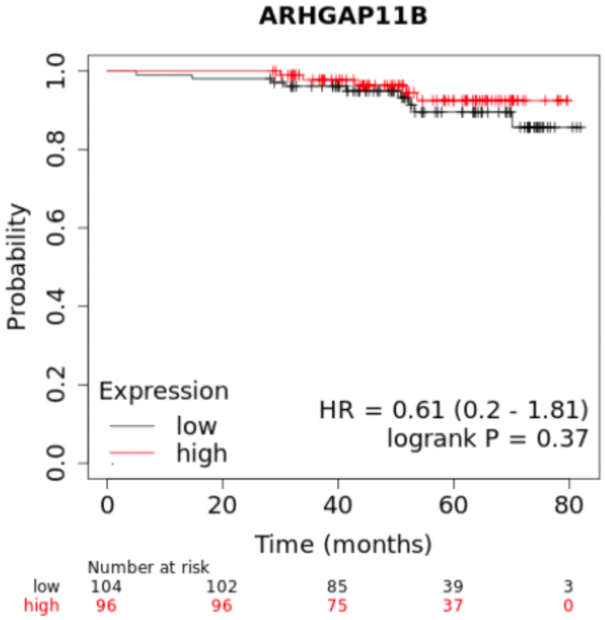

**RNAseq ID:**	ARHGAP11B
**Survival:**	OS
**Auto select best cutoff:**	checked
**Follow up threshold:**	all
**Censore at threshold:**	checked
**Compute median over entire database:**	false
**Cutoff value used in analysis:**	3.03
**Expression range of the probe:**	1 - 4
**Probe set option:**	user selected probe set
**Invert HR values below 1:**	not checked

Restrictions

Lymph node status:	all
ER status:	all
PGR status:	all
HER2 status:	all
KI67 status:	all
Nottingham histologic grade:	all
PAM50 subtype:	Basal

Cohorts

Endocrine treated:	all
Chemo treated:	all

Results

P value: 0.009
FDR: over 50%

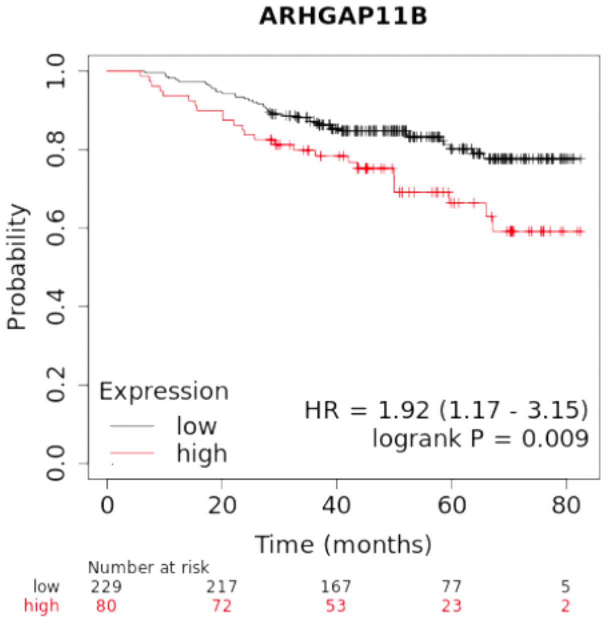

**RNAseq ID:**	ARHGAP11B
**Survival:**	OS
**Auto select best cutoff:**	checked
**Follow up threshold:**	all
**Censore at threshold:**	checked
**Compute median over entire database:**	false
**Cutoff value used in analysis:**	3.24
**Expression range of the probe:**	1 - 5
**Probe set option:**	user selected probe set
**Invert HR values below 1:**	not checked

Restrictions

Lymph node status:	all
ER status:	all
PGR status:	all
HER2 status:	all
KI67 status:	all
Nottingham histologic grade:	all
PAM50 subtype:	LuminalA

Cohorts

Endocrine treated:	all
Chemo treated:	all

Results

P value: 0.0686
FDR: 100%

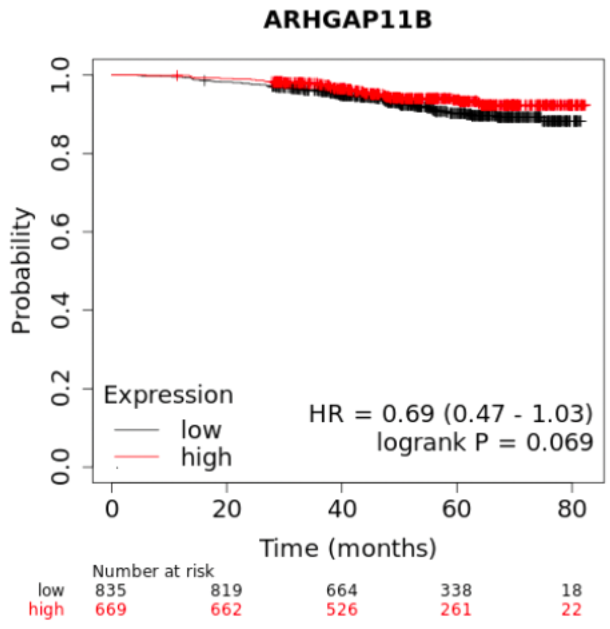

**RNAseq ID:**	ARHGAP11B
**Survival:**	OS
**Auto select best cutoff:**	checked
**Follow up threshold:**	all
**Censore at threshold:**	checked
**Compute median over entire database:**	false
**Cutoff value used in analysis:**	2.77
**Expression range of the probe:**	-1 - 5
**Probe set option:**	user selected probe set
**Invert HR values below 1:**	not checked

Restrictions

Lymph node status:	all
ER status:	all
PGR status:	all
HER2 status:	all
KI67 status:	all
Nottingham histologic grade:	all
PAM50 subtype:	LuminalB

Cohorts

Endocrine treated:	all
Chemo treated:	all

Results

P value: 0.0215
FDR: over 50%

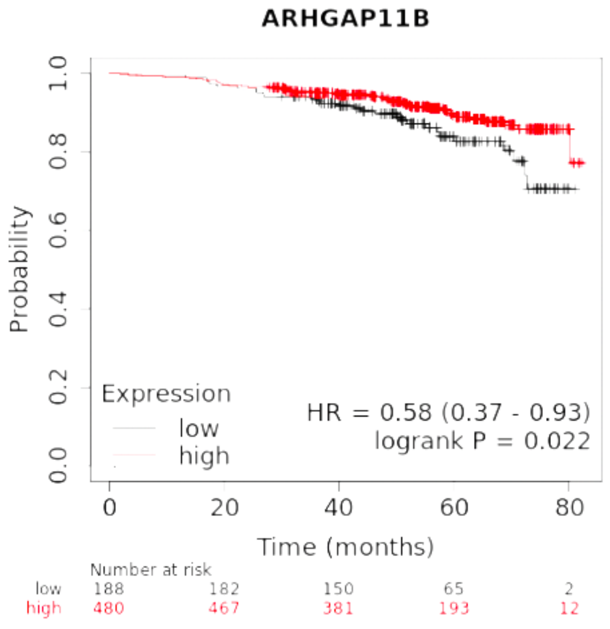

RNAseq ID: ARHGAP11B
Survival: OS
Auto select best cutoff: checked
Follow up threshold: all
Censore at threshold: checked
Compute median over entire database: false
Cutoff value used in analysis: 3.21
Expression range of the probe: 2 - 5
Probe set option: user selected probe set
Invert HR values below 1: not checked

Restrictions

Lymph node status: all
ER status: all
PGR status: all
HER2 status: all
KI67 status: all
Nottingham histologic grade: all
PAM50 subtype: Her2

Cohorts

Endocrine treated: all
Chemo treated: all

Results

P value: 0.0354
FDR: over 50%

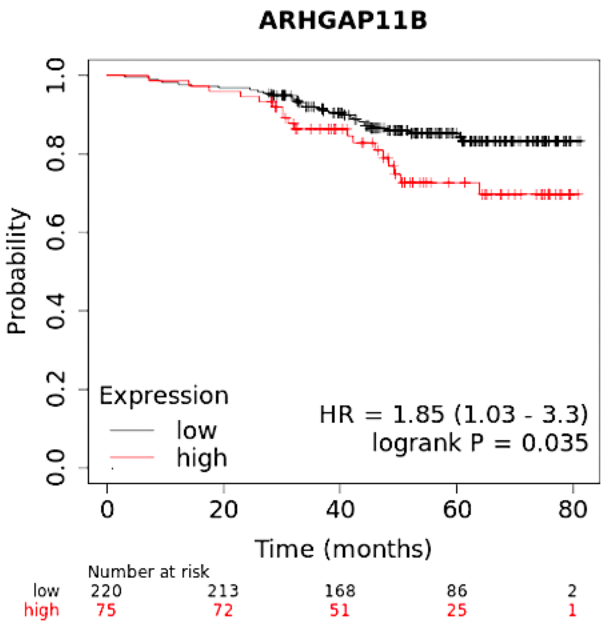

Supplement: Supplementary 7 — File S7: correlation between expression of ARHGAP11B and OS possibility in different types of breast cancer. [file 8236853.f7.pdf]
